# Roles of the TRAPP-II Complex and the Exocyst in Membrane Deposition during Fission Yeast Cytokinesis

**DOI:** 10.1371/journal.pbio.1002437

**Published:** 2016-04-15

**Authors:** Ning Wang, I-Ju Lee, Galen Rask, Jian-Qiu Wu

**Affiliations:** 1 Department of Molecular Genetics, The Ohio State University, Columbus, Ohio, United States of America; 2 Department of Biological Chemistry and Pharmacology, The Ohio State University, Columbus, Ohio, United States of America; Princeton University, UNITED STATES

## Abstract

The cleavage-furrow tip adjacent to the actomyosin contractile ring is believed to be the predominant site for plasma-membrane insertion through exocyst-tethered vesicles during cytokinesis. Here we found that most secretory vesicles are delivered by myosin-V on linear actin cables in fission yeast cytokinesis. Surprisingly, by tracking individual exocytic and endocytic events, we found that vesicles with new membrane are deposited to the cleavage furrow relatively evenly during contractile-ring constriction, but the rim of the cleavage furrow is the main site for endocytosis. Fusion of vesicles with the plasma membrane requires vesicle tethers. Our data suggest that the transport particle protein II (TRAPP-II) complex and Rab11 GTPase Ypt3 help to tether secretory vesicles or tubulovesicular structures along the cleavage furrow while the exocyst tethers vesicles at the rim of the division plane. We conclude that the exocyst and TRAPP-II complex have distinct localizations at the division site, but both are important for membrane expansion and exocytosis during cytokinesis.

## Abstract 2

Two putative vesicle tethers—the exocyst and TRAPP-II complexes—localize differently at the division plane to ensure efficient plasma-membrane deposition along the whole cleavage furrow during cytokinesis in the fission yeast *Schizosaccharomyces pombe*.

## Author Summary

Cytokinesis partitions a mother cell into two daughter cells at the end of each cell-division cycle. A significant amount of new plasma membrane is needed at the cleavage furrow during cytokinesis in many cell types. Membrane expansion is achieved through the balance of exocytosis and endocytosis. It is poorly understood where and when the membrane is deposited and retrieved during cytokinesis. By tracking individual vesicles with high spatiotemporal resolution and using electron microscopy, we found that new membrane is deposited relatively evenly along the cleavage furrow in fission yeast, while the rim of the division plane is the predominant site for endocytosis. The secretory vesicles/compartments carrying new membrane are mainly delivered along formin-nucleated actin cables by myosin-V motors. Surprisingly, we find that both exocytosis and endocytosis at the division site are ramped up before contractile-ring constriction and last until daughter-cell separation. We discovered that two putative vesicle tethers, the exocyst and TRAPP-II complexes, localize to different sites at the cleavage furrow to promote tethering of different, yet overlapping, classes of secretory vesicles/compartments for exocytosis and new membrane deposition.

## Introduction

Cytokinesis partitions a mother cell into two daughter cells following chromosome segregation. In most eukaryotes, except plants, cytokinesis relies on an actomyosin contractile ring, the constriction of which in coordination with plasma-membrane invagination forms the cleavage furrow [1,2]. A significant amount of new plasma membrane is needed for cytokinesis in many cell types [3–5]. Membrane expansion is under sophisticated regulation of the exocytic and endocytic pathways [6–8].

During exocytosis, post-Golgi secretory vesicles mostly travel on actin or microtubule cytoskeleton to their destination [9,10]. Once vesicles approach the target membrane, a series of reactions trigger the vesicle-membrane fusion: tethering, docking, priming, SNARE complex assembly, and fusion [11]. Tethering determines the sites and specificity of vesicle fusion. Vesicle tethers physically attach vesicles to the target membrane over a distance and promote the subsequent fusion processes [12,13]. The pairing between v-SNARE on the vesicle and t-SNARE on the plasma membrane provides the force for the vesicle-membrane fusion [14,15]. On the other hand, branched actin filaments nucleated by the Arp2/3 complex provide the force for membrane invagination during clathrin-mediated endocytosis [16–18]. Given that endocytosis removes membrane from the plasma membrane, it is intriguing that cytokinesis is inhibited or delayed when endocytosis is blocked by drug treatment or in endocytic mutants [19–21]. In mammalian cells, recycling endosomes, which have irregular tubulovesicular shapes [22], deliver retrieved membrane from endocytosis back to the cell surface for reuse [23,24]. Therefore, close investigation of exocytic and endocytic events during cytokinesis is of great interest.

Although studies from different systems have not reached a consensus, it is believed that new plasma-membrane insertion in most animal cells and budding yeast is biased towards the leading edge of the cleavage furrow and that the exocyst complex tethers vesicles delivering the new membrane for cytokinesis [7,25–27]. These are consistent with the unified view of cytokinesis that the contractile ring guides and coordinates membrane invagination [28]. However, these paradigms have not been rigorously tested using live-cell imaging with high spatiotemporal resolution.

The octameric exocyst is the main tether of vesicles at the plasma membrane in all eukaryotes [29–31] and the only known vesicle tether functioning during cytokinesis in animal cells and fungi [32–34]. Besides the exocyst, many other vesicle tethers in two groups are involved in intracellular vesicle trafficking: elongated coiled-coil tethers and a variety of multisubunit tethering complexes (MTCs) including the transport protein particle (TRAPP) complexes [13,35,36]. Initially discovered in budding yeast, the three forms of TRAPP complex (TRAPP-I, -II, and -III) share six core components but have distinct functions [37,38]. The TRAPP-II complex is proposed to regulate intra-Golgi, endosome-Golgi, and Golgi-exit trafficking in budding yeast and plant cells [37,39,40] but has not been shown to function in cytokinesis in yeast or mammalian cells. Interestingly, TRAPP-II is required for cleavage furrow ingression in male meiotic *Drosophila* cells [41], and TRAPP-II and exocyst complexes play sequential and overlapping roles in plant cytokinesis [42]. However, it was untested in these studies whether the TRAPP-II affects vesicle tethering in cytokinesis. Furthermore, the specificity of the vesicles that TRAPP-II potentially tethers or helps to tether to the cleavage furrow was unknown.

The fission yeast *Schizosaccharomyces pombe* is an excellent model organism for studying cytokinesis because the principle mechanisms and the proteins involved are largely conserved from fission yeast to humans [2,43]. In *S*. *pombe*, the contractile ring is assembled through the condensation of cytokinesis nodes, the ring precursors at the cell equator, into a compact ring [44–47]. The compact ring matures by recruiting more cytokinesis proteins during anaphase B and begins constricting after chromosome segregation [44]. Ring constriction is coupled with invagination of the plasma membrane and formation of a trilaminar septum (primary septum sandwiched by two secondary septa). Digestion of the primary septum leads to cell separation after septum matures.

Rod-shaped *S*. *pombe* cells have a diameter of ~3.5 μm and must add ~19 μm^2^ of new membrane to make a cleavage furrow during cytokinesis. A typical secretory vesicle in yeast has a surface area of ~0.02 to 0.03 μm^2^ [48,49]. Thus, new membrane from >600 secretory vesicles is needed for cytokinesis if assuming secretory vesicles are the only membrane source and not considering membrane loss due to endocytosis. How this immense number of vesicles are brought to the division site is unknown. In fission yeast, the exocyst localizes to the rim of the cleavage furrow throughout cytokinesis, thus it is separated from the constricting ring [33,50–52]. It is known that the exocyst targets the secretory vesicles carrying the hydrolytic glucanases to the division site for daughter cell separation [33,50]. However, it remains untested whether vesicle fusion and membrane deposition are limited to the rim of the cleavage furrow by the localization of exocyst.

Here we find that secretory vesicles/compartments are delivered to the cleavage furrow evenly during contractile-ring constriction by actin cytoskeleton and myosin-V motor, rather than predominantly to the leading edge adjacent to the ring or to the rim of the division plane as predicted. In contrast, endocytosis is more active near the rim of the cleavage furrow. In addition, our data also indicate that the exocyst and the TRAPP-II complex have distinct localizations at the division site but both affect vesicle tethering for plasma-membrane expansion and cargo delivery during cytokinesis.

## Results

### Secretory Vesicles Start to Deliver New Membrane to the Future Division Site before Contractile-Ring Constriction

**Fig 1:**
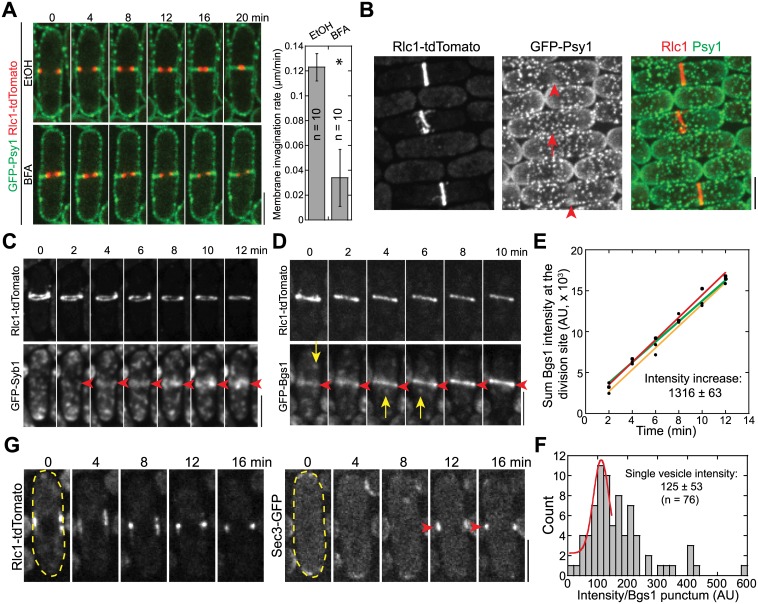
Exocytosis is important for cytokinesis, and vesicles are delivered to the division site during contractile-ring maturation. (A) Time course in min (middle focal plane; left panels) and quantification (right panel) of ring constriction and membrane invagination in cells expressing GFP-Psy1 and Rlc1-tdTomato (strain JW2402, see S1 Table for details) treated with either EtOH or BFA (see Materials and Methods). Time 0 marks the start of the movie. *, *p* < 0.05 compared with the control from two-tailed *t* test in this and other graphs. (B) Psy1 (green) concentrates to the medial cortex during ring (red) maturation, during which the diameter of a compact ring has no changes (indicated by arrowheads, compared to the cell in ring-assembly stage marked by an arrow). (C) Time course of the appearance and localization (arrowheads) of the v-SNARE Syb1 at the division site (sum projection) during ring maturation. (D) Time course (sum projection) and (E) plot (of sum intensity) of accumulation of β-glucan synthase Bgs1 at the division site during ring maturation. Arrowheads mark division-site localization and arrows mark Bgs1 vesicles in (D). Intensity increase rate (mean ± standard deviation [SD]) in min is indicated (*n* = 3 cells). (F) Histogram showing the intensity of single Bgs1 vesicles imaged with the same setting as for the cells in (D, E). Mean ± SD of the first peak (possibly a single vesicle) after fitting with a Gaussian distribution is shown. (G) Time course showing localization of the exocyst subunit Sec3 to the division site during ring maturation (marks with Rlc1 in the same cell) in the middle focal plane. The cell boundaries are marked with broken lines in this and some other figures. Bars, 5 μm.

Consistent with previous studies on exocytosis in fission yeast [33,50], we found that exocytosis is critical for plasma-membrane invagination and contractile-ring constriction during cytokinesis. Brefeldin A (BFA; blocking endoplasmic reticulum [ER] to Golgi trafficking) treatment of wild-type (wt) *S*. *pombe* cells and mutation in exocyst subunit Sec8 [33] both significantly slowed down plasma-membrane invagination (marked with GFP tagged t-SNARE Psy1 [53]) and ring constriction (marked with tdTomato tagged myosin regulatory light chain Rlc1) (Figs 1A, S1A and S1B).

To investigate when the delivery of new membrane to the division site begins during the cell cycle, we examined the localizations of several proteins essential for secretory vesicle tethering and membrane fusion relative to the contractile ring (Fig 1B–1G), including plasma-membrane t-SNARE Psy1, post-Golgi v-SNARE Syb1 [54], a vesicle cargo β-glucan synthase Bgs1 [55], and the exocyst subunit Sec3 [52,56]. Interestingly, additional Psy1 started to accumulate at the cell equator as a weak band (Fig 1B, arrowheads) during contractile-ring maturation (the dwell time after node condensation into a compact ring but before ring constriction [44]), suggesting that vesicle delivery and new membrane deposition start before membrane invagination and ring constriction. Consistently, Syb1 and Bgs1 appeared at the division site and gradually increased in intensity as the ring matured (Figs 1C–1E, S1C and S1D; S1 Video). Based on the intensity of Bgs1 puncta that moved to the division site in a fast and directional manner (Fig 1D, arrows; S1D Fig), we calculated that ≥11 Bgs1 containing vesicles were delivered to the division site per minute during ring maturation (Fig 1E and 1F). Since secretory vesicles with other cargos are delivered to the division site during this time [57], these data suggest that vesicle tethering and deposition begin before cleavage-furrow ingression. Indeed, the exocyst, the only known vesicle tether at the plasma membrane in fungi (see Introduction), localized to the division site right after node condensation into a compact ring (Fig 1G).

### New Membrane Deposition Sites at the Cleavage Furrow Are Not Biased by the Locations of the Exocyst or the Contractile Ring

**Fig 2:**
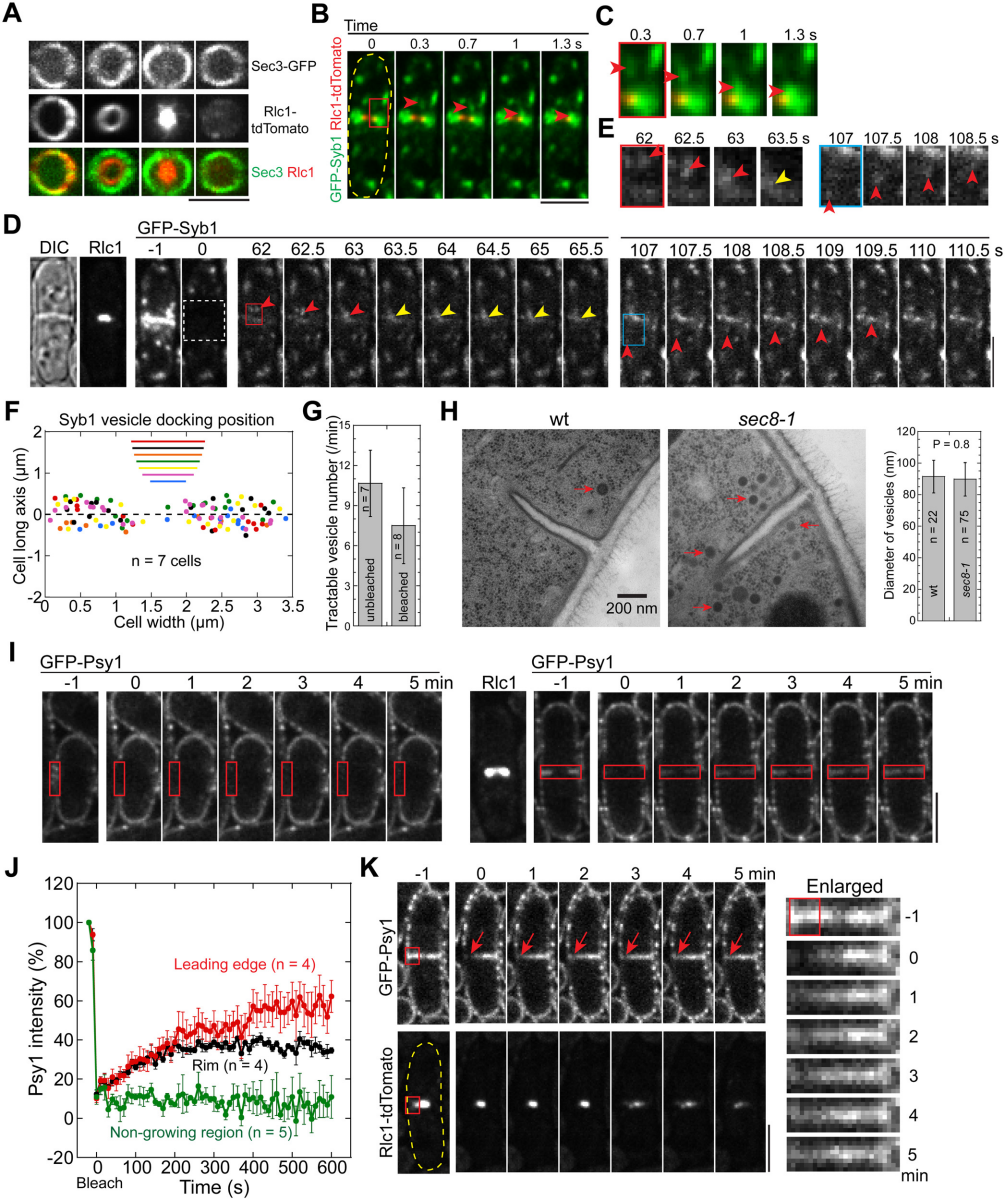
Vesicles and membrane are delivered to the cleavage furrow evenly during ring constriction. (A) Sec3 labeled exocysts stay at the rim of the division plane during ring constriction. The vertical views are projections from z-stacks spaced at 0.2 μm. (B, C) An Syb1 vesicle is deposited just behind the ring at the division plane in the middle focal plane. The traveling vesicles are marked by arrowheads. (C) Enlarged images for boxed region in (B). (D, E) Trafficking of Syb1 vesicles to the division site in a single focal plane after photobleaching Syb1 signal within the white-boxed region in a cell with a partial septa (DIC) and a constricting Rlc1 ring. Two examples of traveling vesicles from the same cell are marked with arrowheads. The yellow arrowheads mark a vesicle remaining still for 1.5 s before signal spreading out, suggesting vesicle tethering/fusion. (E) Enlarged red- and blue-boxed regions in (D). (F) Distribution of final tractable deposition sites of Syb1 vesicles relative to the position of constricting ring (at the beginning of the movie) from 7 cells (color coded) in 2-min movies after Syb1 signal at the division site was bleached as in (D). *X*- and *y*-axes are along the division site and cell long axis, respectively. The ring position (the diameter marked by the color lines) is displaced along the *y*-axis away from its real position at *y* = 0 for clarity. (G) Numbers of tractable Syb1 vesicles per min at the middle focal plane when the division site Syb1 signal is unperturbed or bleached. (H) EM images (left) and diameters (right) of secretory vesicles (arrows) in wt and *sec8-1* mutant. (I-K) Dynamics of Psy1 on the plasma membrane revealed by FRAP. Red boxes mark the bleached regions, which were bleached at time 0. (I) Psy1 dynamics at the non-growing side of an interphase cell (left panel) or the division site of a cell with constricting ring (right panel), which is marked by Rlc1 taken at -1 min. (J) Recovery of Psy1 intensity after photobleaching at time 0. Mean ± standard error of the mean (SEM) is plotted. (K) The center of the bleached region (arrows) at the division site (enlarged on the right) does not move in any directions in cells with a constricting ring. Bars, 5 μm.

We next examined where new membrane is deposited at the cleavage furrow. As reported [33,50–52], the exocyst localized to non-constricting rings at the rim of the cleavage furrow during membrane invagination and ring constriction (Fig 2A), similar to septins [58], which seems to contradict the prevailing model in animal cells that new membrane is predominantly inserted at the leading edge of the cleavage furrow, behind the constricting ring (see Introduction). To elucidate the discrepancy, we tracked the directional movement of v-SNARE Syb1 labeled post-Golgi vesicles towards the division site during ring constriction (Fig 2B–2G; S1 Video, middle panels). Surprisingly, individual punctum could travel to the regions close to the constricting ring (Fig 2B and 2C), to the rim of the division plane where the exocyst localizes, or to other locations along the cleavage furrow (S1 Video, middle panels). Given the heterogeneous shapes and sizes of Syb1 puncta (Fig 2B), Syb1 might label multiple secretory compartments including but not limited to the typical spherical secretory vesicles.

To investigate quantitatively the docking sites for incoming secretory vesicles and/or other Syb1 labeled secretory compartments that travel to the division site, we bleached the Syb1 signal around the division site on a single focal plane and tracked the incoming Syb1 puncta (Fig 2D–2F; S2 Video). Once at the division site, the Syb1 signal often remained still for a few seconds before spreading out (Fig 2D, yellow arrowheads), which may indicate vesicle tethering/fusion. We detected no obvious lateral movement of Syb1 signal along the division plane (S2 Video), indicating that the last tractable location of vesicle was likely the final docking and fusion site. The Syb1 labeled secretory vesicles/compartments were deposited relatively evenly between the constricting ring and the rim of the division plane (Fig 2F), suggesting that the vesicle docking sites at the cleavage furrow are not biased by the locations of exocyst or contractile ring.

We were able to track ~11 Syb1 puncta travelling to the division site per minute on a single focal plane when the division-site Syb1 signal was unperturbed and eight when bleached (Fig 2G). Typical spherical secretory vesicles in wt and exocyst mutant *sec8-1* had similar diameters (90 ± 10 nm) under electron microscopy (EM) (Fig 2H). Their calculated surface area is ~0.025 μm^2^. Thus, ≥0.25 μm^2^ new membrane from the secretory vesicles is delivered to the division site within single focal plane during ring constriction if we assume all the travelling Syb1 puncta are spherical secretory vesicles. We also detected elongated, tubulovesicular membrane structures close to the plasma membrane in EM thin sections (S2A Fig, arrowheads), which resemble recycling endosomes in mammalian cells [22] and the late-Golgi cisternae in budding yeast [59]. The heterogeneity of the shape, size, and intensity of delivered Syb1 puncta (Fig 2B) suggest these tubulovesicular structures also contribute to cytokinesis.

t-SNARE on the target membrane is required for the fusion of a vesicle with v-SNARE through SNARE complex assembly [14,60]. New t-SNARE is also needed for plasma-membrane expansion at the cleavage furrow through vesicle trafficking [61–63]. We investigated the dynamics of the t-SNARE Psy1 on the plasma membrane using fluorescence recovery after photobleaching (FRAP). As predicted from other t-SNAREs [64–66], Psy1 signal barely recovered at the non-growing plasma membrane after photobleaching (Fig 2I, left; Fig 2J; S3 Video, upper panels), indicating the turnover of Psy1 from the membrane is slow. By contrast, Psy1 signal at the division site recovered evenly along the cleavage plane in a few minutes (Fig 2I, right; Fig 2J; S3 Video, lower panels), which is most likely via new membrane insertion. Interestingly, when the Psy1 signal within a small region of the division site was bleached, the center of the bleached region did not move sideways following ring constriction (Fig 2K), suggesting that the lateral movement of plasma membrane exerted by polar expansion [67] is not the main source of membrane in *S*. *pombe* cytokinesis. Thus, our Psy1 data also support that new membrane is inserted evenly at the cleavage furrow.

We also tracked Syb1 vesicle movement and Psy1 dynamics during septum maturation, the stage that the ring has fully constricted and disassembled but before daughter-cell separation (S2B–S2D Fig). Interestingly, a large amount of post-Golgi vesicles/compartments still delivered relatively evenly to the division plane (S2B and S2C Fig). Consistently, Psy1 signal recovered evenly after the division-site signal was bleached (S2D Fig). Together, vesicles are delivered to the division site throughout cytokinesis until cell separation.

EM images of wt cells were also consistent with the even deposition of new membrane during cytokinesis. Secretory vesicles or the tubulovesicular structures appeared to be tethered to the plasma membrane (distance of vesicles to plasma membrane was within ~100 nm) along the whole cleavage furrow instead of exclusively at the leading edge or at the rim (S2A Fig, arrows for vesicles and arrowheads for tubulovesicular structures). Together, our data indicate that new membrane deposition sites at the cleavage furrow are not biased by the locations of the exocyst or the contractile ring.

### For3-Nucleated Actin Filaments and the Myosin-V Myo52 Are Important for Vesicle Trafficking to the Division Site

The fast and directional movements of Syb1 and Bgs1 vesicles (Figs 1, 2, S1 and S2) suggest that they are transported by motors on cytoskeletal tracks. In animal and plant cells, vesicles delivered on microtubules are an important source for new plasma membrane during cytokinesis [25,68]. In budding yeast, myosin-V delivers secretory vesicles on actin cables to the bud for polarized growth and to bud neck for cytokinesis [69–71]. In fission yeast, although both actin filaments and microtubules are involved in polarity establishment and maintenance [72–74], it is unknown which one is more important for vesicle transport to the division site for membrane expansion during cytokinesis.

**Fig 3:**
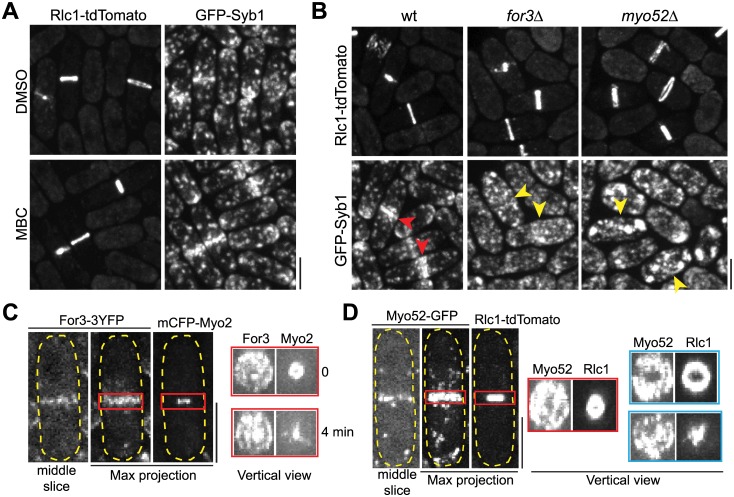
Actin filaments nucleated by the formin For3 and myosin-V motor Myo52 are important for vesicle delivery to the division site. (A) Syb1 localization at the division site does not depend on microtubules revealed by MBC treatment. (B) Syb1 localization at the division site depends on For3 and Myo52. Yellow arrowheads mark examples of greatly reduced Syb1 signal at the division site in mutant cells compared with wt cells (red arrowheads). (C, D) For3 and Myo52 localize to the whole cleavage furrow as a washer or disk during ring constriction (labeled with myosin-II heavy chain Myo2 or light chain Rlc1). Localization of For3 or Myo52 in the middle focal plane, maximum-intensity projection of a z-stack spaced at 0.2 μm, or vertical view of the red-boxed regions of the same cell (and 4 min later for For3) or from another two Myo52 cells with different extent of ring constriction (blue boxes). Bars, 5 μm.

We first investigated whether microtubules affect the delivery of Syb1 vesicles during cell division (Fig 3A). Treatment of wt cells with methyl benzimidazole-2-yl carbamate (MBC) to disrupt microtubules had no detectable effects on Syb1 localization at the division site (Fig 3A). In contrast, Syb1 did not concentrate at the division site during ring constriction in ~93% cells with a deletion of the formin For3 (Fig 3B, yellow arrowheads; *n* = 58 cells), which nucleates actin filaments in actin cables [74]. The myosin-V motor Myo52 is critical for transporting β-glucan synthase Bgs1 and other cargos to cell tips in interphase and to the division site in dividing cells [75,76]. As expected, Syb1 concentration at the division site was abolished in ~90% *myo52*Δ cells during ring constriction (Fig 3B, yellow arrowheads; *n* = 67 cells), indicating that Myo52 is a motor that drives the actin-dependent movement of secretory vesicles/compartments to the division site. The fact that *for3*Δ cells still finish cytokinesis with a delay in ring constriction [77] and *myo52*Δ cells displayed prolonged ring constriction and septum maturation (S3A and S3B Fig) is consistent with the finding that vesicles can reach their destination by actin-independent random walk [56,78]. Thus, For3 nucleated actin cables are critical for efficient delivery of secretory vesicles/compartments by myosin-V motors to the division site.

Next we observed For3 and Myo52 distribution at the division site. We and others found that both For3 and Myo52 dynamically localized to the division plane during cytokinesis [72,75,77,79], forming a disk structure, although the signals were not smooth and continuous (Fig 3C and 3D). This motivated us to track Myo52 movements towards the division plane at different cytokinesis stages. Myo52 puncta docked evenly along the division plane during ring constriction and septum maturation (S3C–S3E Fig; S4 Video, middle and right panels), resembling v-SNARE Syb1 (Figs 2F and S2C). Together, these data support our hypothesis that vesicle tethering and fusion with plasma membrane happen all over the division plane.

### Endocytosis Is More Active at the Rim of the Cleavage Furrow during Cytokinesis

**Fig 4:**
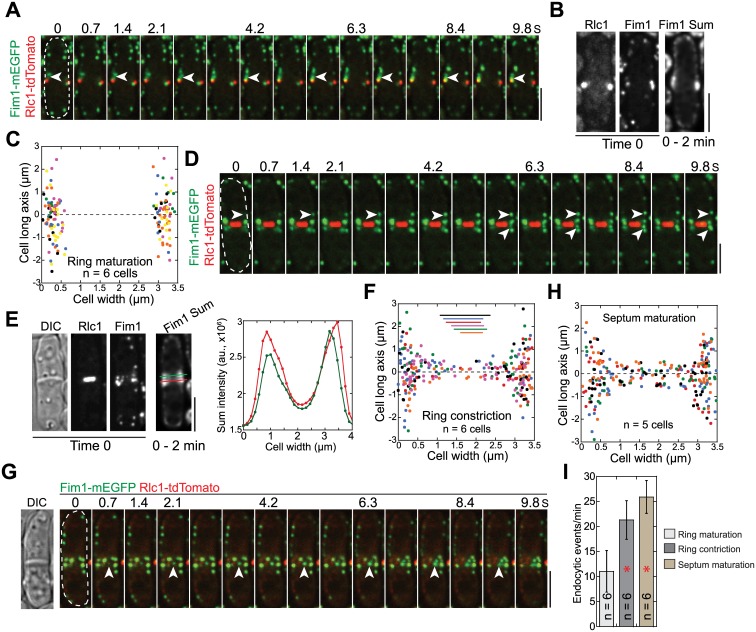
Sites of endocytosis at different stages of cytokinesis. Formation of endocytic patches labeled with fimbrin Fim1 at the division site during ring (Rlc1) maturation (A–C), ring constriction (D–F), and septum maturation (G–H). (A, D, G) Montages showing endocytic patch assembly (arrowheads) viewed in the middle focal plane. (B, E) Sum intensity projection of 498 Fim1 images in the middle focal plane in a 2-min movie along with Rlc1, Fim1, and DIC images at time 0. The right panel in E: line scans (color-coded) of Fim1 intensity as marked on the sum image to the left. (C, F, H) Distribution of endocytic patch assembly sites from multiple cells (color-coded) during ring maturation, ring constriction, and septum maturation. The dashed line at *y* = 0 marks the division site. (F) The ring (the diameter marked by the color lines) is displaced along the *y*-axis away from its real position at *y* = 0 for clarity. (I) Numbers of assembled Fim1 patches in the middle focal plane per minute at different stages of cell division. *, *p* < 0.05 compared with ring maturation stage. Bars, 5 μm.

The balance between exocytosis and endocytosis controls plasma-membrane expansion during polarized growth and cytokinesis (see Introduction). Thus, we investigated how endocytosis contributes to cytokinesis, which was unclear partly due to insufficient investigation into the precise locations of endocytosis during cell division. In *S*. *pombe*, active endocytic sites are at growing cell tips during interphase and at the cell equator during cytokinesis [80]. The appearance of actin cross-linker fimbin Fim1 at endocytic patches represents the growth of actin filaments surrounding the endocytic pits [81,82], which can be used to mark the endocytic sites. Using Rlc1 to label cytokinesis nodes and the contractile ring, we found that endocytic patches started to assemble at the cell equator during ring maturation (Fig 4A–4C and S5 Video), which is consistent with the timing of the relocation of other proteins involved in endocytosis from cell tips to the division site [80,83–85].

Next we examined the distribution of endocytic sites during cell division using the emerging locations of Fim1 patches. During ring maturation, the predominant site for endocytic activity appeared as a broad band on the plasma membrane at the cell equator around the contractile ring (Fig 4B and 4C). During ring constriction, Fim1 emerged from both the cortex adjacent to the division plane (within ~2 μm) and the cleavage furrow (Fig 4D–4F). However, more endocytosis occurred on or near the rim of the cleavage furrow (Fig 4E and 4F). Endocytosis was even more active in later stage of cytokinesis when a full septum has formed (Fig 4G and 4H; S5 Video). During this stage, more endocytic events were detected (Fig 4I) and slightly more endocytic patches formed at the interior of the division plane (Fig 4H). Together, frequencies and locations of endocytic events appeared to be temporally regulated.

### Putative Vesicle Tether TRAPP-II Complex Localizes to Trans- and Post-Golgi Secretory Vesicles/Compartments and the Division Site during Cytokinesis

**Fig 5:**
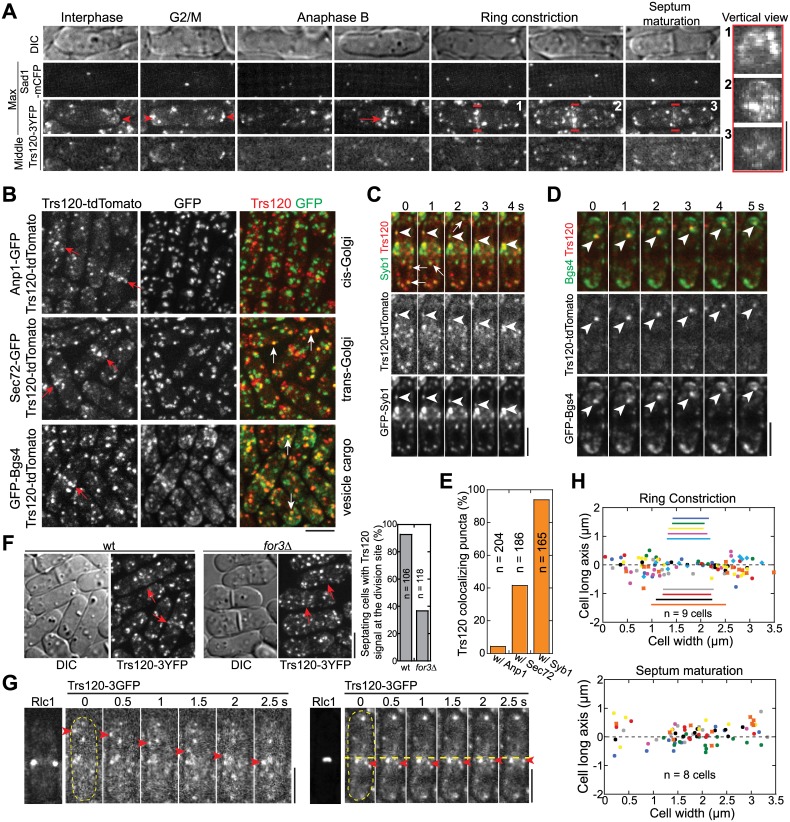
The TRAPP-II complex colocalizes with post-Golgi vesicles and travels to the division site during cytokinesis. (A) Trs120 localization to cell tips (arrowheads) and division site (arrow) during the cell cycle marked with spindle pole body protein Sad1. Images of DIC, max intensity projection, and middle focal plane of cells are shown along with vertical views on the right of the regions marked by short red lines on the numbered cells. (B) Trs120 colocalizes with Sec72 and Bgs4 (examples marked by white arrows) but not with Anp1 labeled cis-Golgi structures. Red arrows point out the division-site signal of Trs120. (C) Trs120 colocalizes with Syb1 labeled vesicles/compartments (arrows) and travels to the division site with Syb1 (arrowheads) during cytokinesis. (D) A Trs120 punctum travels with Bgs4 to the cell tip in an interphase cell. (E) Percentage of Trs120 puncta containing Anp1, Sec72, or Syb1. (F) Trs120 accumulation at the division site (arrows) is dramatically reduced or undetectable in *for3*Δ. Quantification of septating cells (with partially or fully formed septa) with detectable Trs120 concentration at the division site is shown on the right. (G) Trs120 puncta (arrowheads) move to the division site during ring maturation (left) and constriction (right). The dashed horizontal line is to aid tracking Trs120. (H) Distribution of final tractable docking sites of Trs120 labeled puncta in 2-min movies during ring constriction (top) and septum maturation (bottom). The data are plotted as in Fig 4F and 4H. Bars, 5 μm.

Our finding that new membrane is deposited along the whole cleavage furrow (Figs 2 and S2) but the exocyst concentrates at the rim of the division plane (Fig 2A; [33,50,51,56]) suggests that other vesicle tethers or tethering factors contribute to cytokinesis besides the exocyst. The TRAPP-II complex has been proposed to regulate cytokinesis in plants and *Drosophila* [41,42]. Thus, we tested whether TRAPP-II is involved in cytokinesis to help tethering vesicles in fission yeast. Trs120, a specific component of the TRAPP-II complex [86], localized to punctate structures (Fig 5A–5D) and was slightly concentrated at the growing cell tips in interphase cells (Fig 5A, arrowheads). In late anaphase B, a fraction of Trs120 puncta concentrated to cell equator, the future division site (Fig 5A, arrow; S6 Video). During ring constriction and septum formation, Trs120 puncta localized along the division plane (Fig 5A, vertical views), similar to For3 and Myo52 (Fig 3C and 3D).

Trs120 puncta resemble secretory vesicles, we reasoned that they might participate in vesicle trafficking. Indeed, Trs120 puncta partially co-localized with the trans-Golgi network marker Sec72 [87] and secretory vesicles/compartments (β-glucan synthase Bgs4, a vesicle cargo [57]; Fig 5B, white arrows; Fig 5E), but did not overlap with cis-Golgi (Golgi mannosyltransferase complex component Anp1 [87]; Fig 5B and 5E) or transitional-ER (COPII vesicle coat protein Sec24 [87]). ~94% of Trs120 puncta also colocalized with Syb1 labeled trans- or post-Golgi vesicles/compartments (Fig 5C, arrows; Fig 5E) and traveled with Syb1 to the division site during cytokinesis (Fig 5C, arrowheads) although only ~50% Syb1 puncta contained Trs120 (*n* = 179 puncta). Trs120 may also be involved in cargo delivery to cell tips during interphase, given that Trs120 and Bgs4 moved together on some puncta to cell tips (Fig 5D, arrowheads). The division-site accumulation of Trs120 largely depended on the formin For3 (Fig 5F).

We next tracked the movement of Trs120 puncta towards the division site at different stages of cytokinesis (Fig 5G and 5H; S7 Video). During ring constriction, Trs120 puncta were delivered to the whole cleavage furrow, with a slight bias towards the region immediately behind the ring (Fig 5H). During septum maturation, Trs120 puncta were mainly delivered to the center of the division plane (Fig 5H). Consistently, sum projections of the 2-min movies indicated that Trs120-3GFP was more concentrated at the leading edge of the cleavage furrow (S4A Fig, arrows). Interestingly, Trs120 puncta only stayed at the division site for ~8 s before the signal disappeared (S4B and S4C Fig; S7 Video). Thus, Trs120 is dynamic at the division site and likely leave the plasma membrane after/during vesicle fusion. Together, the TRAPP-II complex travels with vesicles/compartments to the division site and may help to tether them to the plasma membrane during cytokinesis.

### TRAPP-II Mutants Display Cytokinesis Defects and Synthetic Genetic Interactions with Exocyst Mutants

**Fig 6:**
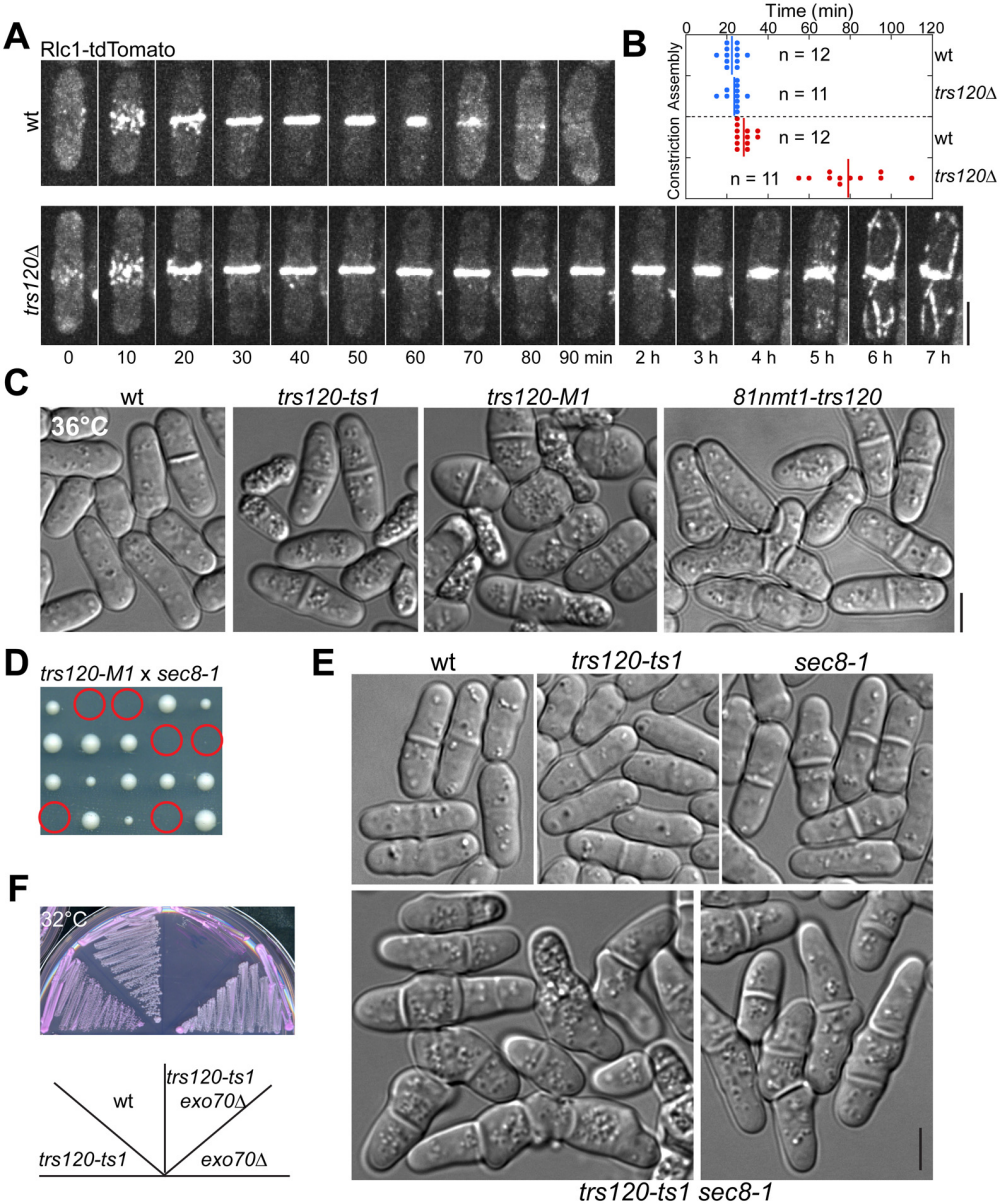
Phenotypes of TRAPP-II mutants and synthetic interactions between TRAPP-II and exocyst mutations. (A, B) Time courses (A) and quantification (B) of the contractile-ring assembly (from the appearance of cytokinesis nodes to formation of a compact ring without lagging nodes) and constriction (from the start of ring contraction until it constricts to a dot with the highest Rlc1 intensity) in wt and *trs120*Δ cells observed with tetrad fluorescence microscopy. (B) The rings of ~50% *trs120*Δ cells did not or only partially constricted during the 14-h movies, so the quantification includes only those fully constricted ring. (C) Morphological defects of three *trs120* mutants in YE5S medium at 36°C. Wt, *trs120-ts1*, *trs120-M1*, and *81nmt1-trs120* cells were cultured at 36°C for 6, 4, 2, and 6 h, respectively. (D, E) Synthetic genetic interactions between TRAPP-II and exocyst mutations. (D) The predicted *trs120-M1 sec8-1* mutant (circles) cannot form colonies on YE5S plate at 25°C. (E) DIC images showing the synthetic interaction between *trs120-ts1* and *sec8-1* mutations at 25°C. (F) Growth of *trs120-ts1*, *exo70*Δ, and the double mutant at 32°C on YE5S medium with phloxin B (PB), which accumulates in dead cells [131]. Bars, 5 μm.

To further study the function of TRAPP-II complex in cytokinesis, we examined the phenotype of *trs120* mutants. Since *trs120* is an essential gene [88], we generated a homozygous diploid strain expressing integrated Rlc1-tdTomato and then deleted one copy of *trs120*. We performed tetrad fluorescence microscopy [77,89,90] to observe the growth and division of wt and *trs120*Δ haploid cells after the *trs120* ^+^/*trs120*Δ diploid underwent meiosis and sporulation. Haploid cells with *trs120* deleted usually died after two to four generations. *trs120*Δ cells had no obvious defects in contractile-ring assembly (Fig 6A and 6B), but often failed (Fig 6A, *n* = 12 cells) or had very slow (Fig 6B, *n* = 11 cells) ring constriction and plasma-membrane invagination, which led to premature ring disassembly, incomplete membrane insertion, and/or cell lysis. This indicates that the TRAPP-II is involved in later stages of cytokinesis.

To facilitate rapid inactivation of TRAPP-II complex in vivo, we generated temperature-sensitive *trs120* mutants using a marker reconstitution mutagenesis method [89,91]. Both *trs120-ts1* and *trs120-M1* mutants had cytokinesis defects at 36°C with increased septation indices, formation of multi-septated cells, and cell lysis (Fig 6C). *trs120* depletion mutant *81nmt-trs120* showed similar phenotype at 36°C (Fig 6C). Consistent with our hypothesis that the exocyst and TRAPP-II complexes have overlapping functions in vesicle trafficking, exocyst mutant *sec8-1* [33,49] was synthetic lethal with *81nmt-trs120* and *trs120-M1* (Fig 6D). *trs120-ts1 sec8-1* double mutant, although viable, displayed severe cytokinesis defects such as multi-septated cells at 25°C (Fig 6E) and stopped growing at 30°C (S4D Fig). *trs120-ts1*, *trs120-M1*, and *81nmt-trs120* also displayed additive growth defects with exocyst mutant *exo70*Δ (Fig 6F). Given that the two complexes had different localizations during ring constriction, septum formation and maturation (Figs 2A, 5A, S4A and S4B; S6 and S7 Videos), it is not surprising that Trs120 localization appeared normal in exocyst mutant *sec8-1* (S4E Fig) and the localization of exocyst component Sec3 was not perturbed by *trs120* mutations (S4F and S4G Fig). Together, the TRAPP-II and exocyst are independent for localization to the division site but have overlapping functions during late stages of cytokinesis.

### TRAPP-II and Exocyst Mutants Affect the Delivery of Cargo Proteins to the Division Site in Distinct Patterns

**Fig 7:**
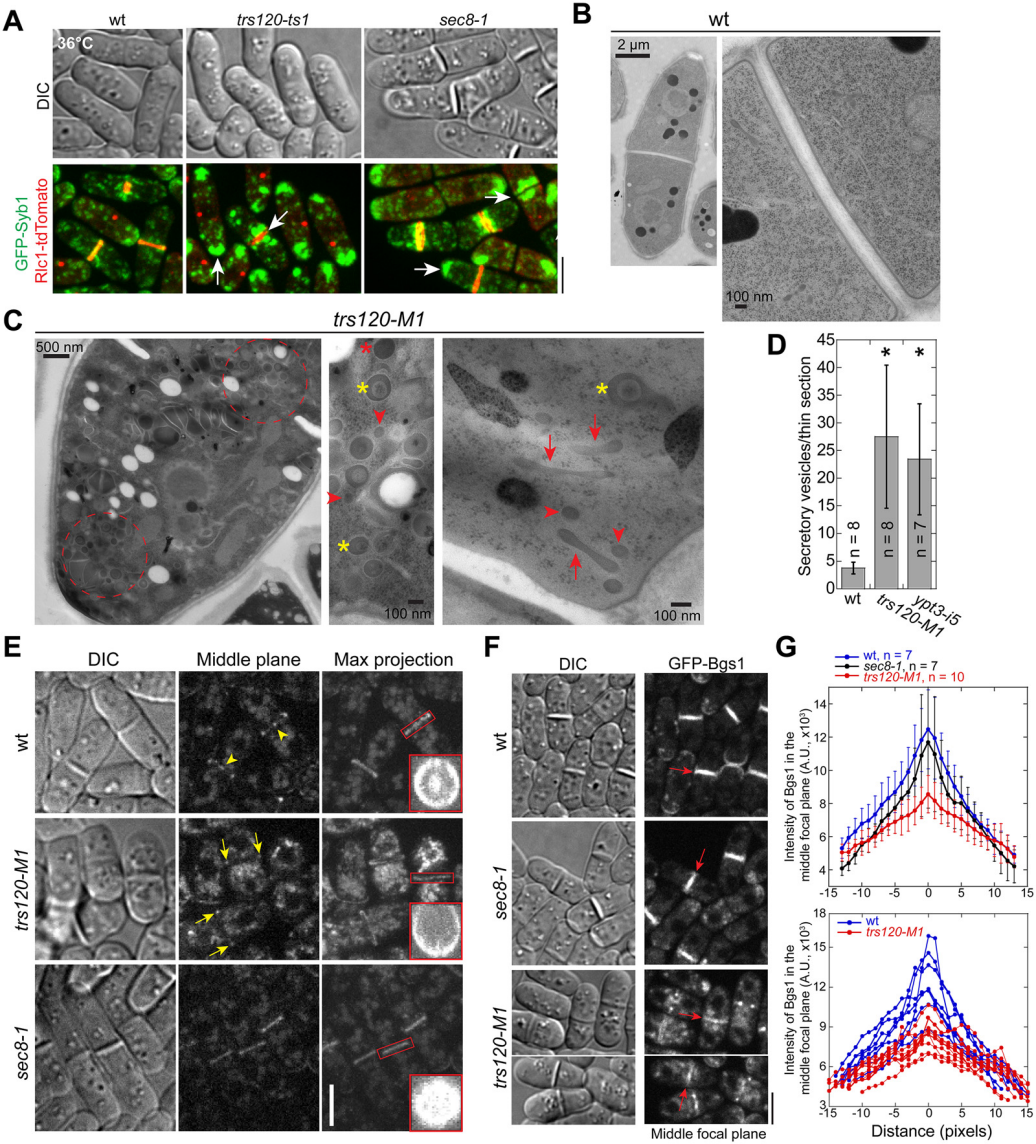
Exocyst and TRAPP-II mutants affect cargo delivery to the division site differently. (A) Syb1 accumulates at the division site or cell tips in *trs120-ts1* and *sec8-1* mutants grown in YE5S medium at 36°C for 2 h. (B–D) EM images (B, C) and quantification (D) showing abnormal accumulation of vesicles and other secretory compartments at the division site or cell tips in *trs120-M1* or *ypt3-i5* cells grown at 36°C for 4 h. (D) Quantification of secretory vesicles or round-shaped vesicle-like structures (diameter <150 nm without engulfed membrane-bound materials) accumulated in each longitude EM thin section. (E) Localization of the glucanase Eng1 to the division site in *trs120-M1* and *sec8-1* cells. Arrowheads mark the center localization of Eng1 at the division plane in wt cells. Arrows mark the remaining Eng1 at the rim in *trs120*-*M1*. Vertical views of the boxed regions are shown at the bottom corner. (F, G) Localization of glucan synthase Bgs1 in the middle focal plane at the division site in *trs120-M1* and *sec8-1* cells during septum maturation. (G) Line scans of Bgs1 intensity along the division plane (marked by arrows in F). Top, Mean ± SD from multiple cells. Bottom, individual wt and *trs120-M1* cells. Bars in A, E, F, 5 μm.

To test whether TRAPP-II mutants are defective in exocytosis, we first examined the localization of v-SNARE Syb1 and the secretion of acid phosphatase. Similar to *sec8-1*, *trs120-ts1* mutant accumulated Syb1 in proximity to the division plane in dividing cells and at the cell tips in interphase cells (Fig 7A). The secretion of acid phosphatase was reduced in two *trs120* mutants although not as dramatic as in *sec8-1* (S4H Fig), suggesting that TRAPP-II functions in exocytosis.

Secretory vesicles accumulate in exocyst mutants (Fig 2H; [33]). We next observed *trs120* mutant using EM to confirm the defects in exocytosis (Fig 7B–7D). Vesicles with a diameter ~90 nm (the typical size of post-Golgi secretory vesicles) accumulated at the division site during cytokinesis or at the cell tips during interphase (circled regions) in *trs120-M1* mutant cells (Fig 7C, arrowheads; Fig 7D). We also noticed accumulation of tubulovesicular membrane structures in *trs120-M1* cells similar to the tubular structures we observed previously in wt cells (Fig 7C, arrows; S2A Fig), which may be trans-Golgi cisternae or early/recycling endosomes. In addition, many large organelles (150–300 nm) either with or without engulfed membrane-bound materials (Fig 7C, yellow and red asterisks, respectively) were detected, likely representing lysosomes or late endosomes. TRAPP-II has been suggested to affect several steps in secretory pathway [37,39,40,86], which may explain the detection of various vesicular membrane structures in the *trs120* mutant.

Given the different localization of exocyst and TRAPP-II at the division site (Figs 2A, 5A, S4A and S4B) and the accumulation of distinct secretory vesicles as well as the tubulovesicular membrane structures in their mutants (Figs 2H and 7C), the exocyst and TRAPP-II complex may affect recruiting vesicles with different cargos to distinct sites on the plasma membrane. To test this idea, we observed the localization of the glucanase Eng1 and the glucan synthase Bgs1, two post-Golgi cargos [55,92], in *sec8-1* and *trs120-M1* mutants. Eng1 localizes to the rim of the division plane as a ring and to the center as a bright spot (arrowheads) in wt cells during septum maturation (Fig 7E; [93]). As reported [50], Eng1 signal spread throughout the division plane with less signal at the rim in *sec8-1* mutant (Fig 7E), although total Eng1 intensity at the division site appeared normal. In *trs120-M1* mutant, the Eng1 intensity at the division plane seemed to be lower than in wt and *sec8-1* and the central spot localization was lost in 93% cells during septum maturation (Fig 7E; *n* = 41 cells). These data suggested that TRAPP-II is more important for cargo delivery to the center, whereas the exocyst is more important for delivery to the rim of the division plane. Consistently, *trs120-M1* mutation decreased Bgs1 intensity at the center of the cleavage furrow dramatically, whereas *sec8-1* compromised Bgs1 localization at the rim (Fig 7F and 7G). Together, our data indicate that the exocyst and TRAPP-II complexes may function at distinct spatial locations to tether (or help tether) vesicles for membrane and cargo delivery during cytokinesis.

### The TRAPP-II Complex Works Preferentially with the Rab11 GTPase Ypt3 in Cytokinesis

TRAPP complexes have been proposed to work with Rab GTPases to tether vesicles by functioning as their guanine nucleotide exchange factors (GEFs) [37,94,95]. Recently, the TRAPP-II was shown to activate Rab11 as a GEF in *Aspergillus nidulans* [40]. Mammalian Rab11 GTPase is a marker for recycling endosomes that are critical for transporting internalized proteins from endocytosis back to cell surface [96,97]. In budding yeast, the recycling traffic to cell surface is through late Golgi [98–100], and Rab11 GTPases Ypt31/32 label late-Golgi cisternae and the secretory vesicles emerging from late Golgi in a signaling cascade to activate Sec4, the Rab8 GTPase on secretory vesicles for polarized exocytosis [101,102].

**Fig 8:**
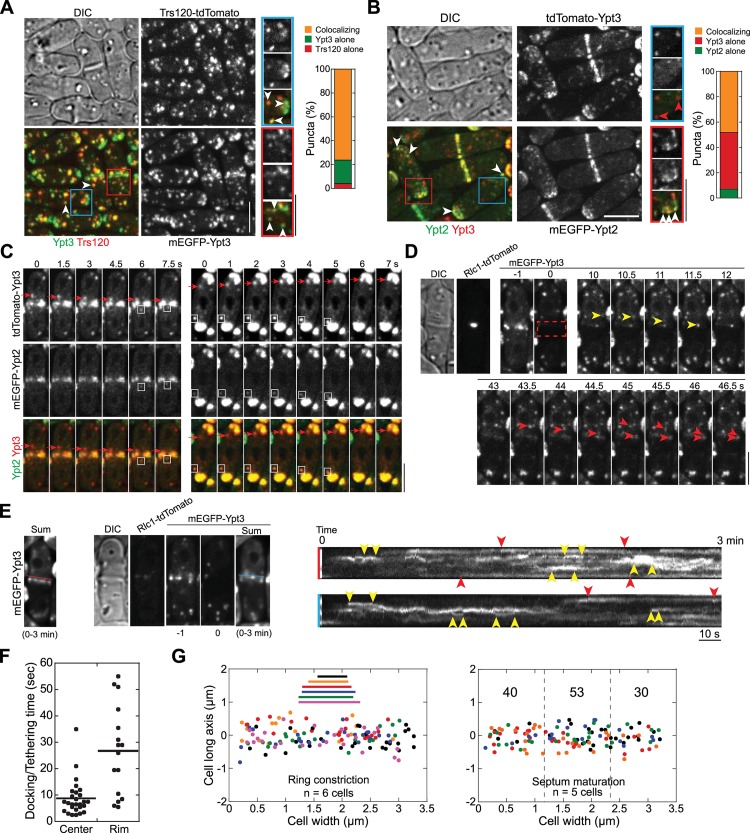
Trs120 co-localizes with Rab11 GTPase Ypt3 and the TRAPP-II complex works preferentially with Ypt3 in cytokinesis. (A, B) Micrographs (left) and quantification (right) of co-localization (arrowheads) of Ypt3 with Trs120 (A, *n* = 439 puncta) or Rab8 GTPase Ypt2 (B, *n* = 141 puncta) in the puncta. Best focal planes for the boxed regions are enlarged and shown on the right. (C) Ypt3 puncta travel to the division site during cytokinesis and to cell tips during interphase alone (arrows) or with Ypt2 (boxes) imaged in single focal plane. (D-G) Analyses of trafficking and docking of Ypt3 labeled secretory vesicles/compartments to the division site after photobleaching Ypt3 signal within the boxed region at time 0 as illustrated in (D). (D) Time course of Ypt3 trafficking during ring constriction in a single focal plane. The yellow and red arrowheads mark a vesicle travelling to the leading edge and the rim of the cleavage furrow, respectively. (E) Left, sum projection of the 3-min movie for the cell in (D). Middle panels, sum projection of Ypt3 signal for another cell during septum maturation along with Rlc1 and DIC images at time 0 and Ypt3 images at time -1 and 0 min. Right panels, kymographs along the color-coded lines marked on the sum projections on the left (to avoid blocking Ypt3 signal, the lines were moved upward slightly) showing different docking/tethering times for Ypt3 puncta at the leading edge (or center) or the rim of the division site. Pairs of red and yellow arrowheads mark Ypt3 with long and short dwell time, respectively. (F) Docking/tethering time for Ypt3 puncta at the center or the rim of the division site of 10 cells during late stage of ring constriction or early stage of septum maturation. (G) Distribution of final tractable docking sites of Ypt3 labeled puncta in 3-min movies during ring constriction (left) and septum maturation (right, numbers of vesicles/compartments delivered to each region are shown). The data are plotted as in Fig 4F and 4H. Bars, 5 μm.

We tested whether the TRAPP-II complex works with Rab11 homologue Ypt3 [103] or Rab8 homologue Ypt2 [104] in *S*. *pombe*. Trs120 and Ypt3 co-localized in ~80% cytoplasmic puncta (*n* = 439 puncta from 8 cells) although more Ypt3 was on the plasma membrane (Fig 8A). However, only 14% of Trs120 puncta contained with Ypt2 (S5A Fig, arrowheads; *n* = 159 from 8 cells). Interestingly, Ypt2-containing puncta in the cytoplasm seemed to be more homogeneous than Ypt3 puncta (Fig 8B). Ypt2 and Ypt3 colocalized in ~48% of the punctate structures, mainly at cell tips or the division site (Fig 8B, white arrowheads), suggesting that they act in a similar signaling cascade on the secretory vesicles as in budding yeast [101]. However, ~45% of puncta contained only Ypt3 but no Ypt2 (Fig 8B, red arrowheads), suggesting that Ypt3 also functions independent of Ypt2 GTPase. Indeed, those Ypt3-only puncta also traveled to the division site during cytokinesis and to the growing cell tips during interphase (Fig 8C and S5B Fig, arrows). Given that ~70% of Ypt3 puncta (derived from S5C Fig) colocalized with the cargo β-glucan synthase Bgs4 (S5C Fig, arrowheads), these Ypt3-only secretory compartments may also be important for cargo delivery and membrane traffic, and likely resembled the tubulovesicular structures detected in EM images (Figs S2A and 7C). Together, our data suggest that the TRAPP-II mainly works on Rab11 Ypt3-labeled secretory compartments regardless of whether Ypt2 is present.

Next we tracked the movement of Ypt3-labeled secretory vesicles/compartments towards the division site during cytokinesis (examples in Figs 8D, S5D and S5E). Similar to Trs120, Ypt3 puncta were targeted to the division site during ring maturation (S5E Fig, arrows; S8 Video). The rim and the leading edge of the division site had slightly more Ypt3 during ring constriction and septum maturation (Fig 8E). However, tracking of individual Ypt3 puncta suggested that Ypt3 puncta at the rim docked for longer time before signal diffused on the plasma membrane (Fig 8E, red arrowheads; S5D Fig, red squares) than Ypt3 puncta delivered to the center of the cleavage furrow (Fig 8E, yellow arrowheads; S5D Fig, yellow squares). Ypt3 vesicles delivered to the center were docked/tethered for ~9 s (Fig 8F), similar to the time that Trs120/TRAPP-II stayed at the division plane (S4C Fig). The docking/tethering time for Ypt3 at the rim was ~27 s (Fig 8F), close to the reported tethering time of the exocyst in budding yeast (~18 s; [105]). Ypt3-labeled secretory vesicles or recycling endosome equivalents were delivered to the whole division plane likely with a small bias towards the center of the maturing septum (Fig 8G; S9 Video), similar to Trs120 (Fig 5G and 5H). Thus, TRAPP-II may specifically recognize Ypt3 containing secretory structures throughout the cleavage furrow.

Additional lines of genetic and cellular evidence further support that the TRAPP-II complex and Rab11 GTPase Ypt3 work together in cytokinesis. First, similar to its homolog in *A*. *nidulans* [40], TRAPP-II is important for Ypt3 localization since cell tip and division-site localization of Ypt3 was dramatically reduced in *trs120* mutants, although Ypt3 global level was not affected by the *trs120* mutation (S6A Fig). Second, Trs120 was more concentrated at the division site (red arrow) and cell tips (yellow arrow) in *ypt3-i5* mutant (S6B Fig), possibly due to delayed fusion of TRAPP-II containing vesicles to the plasma membrane. Third, *ypt3-i5* mutant accumulated GFP-Syb1 at the active growth sites (S6C Fig, arrows), similar to *trs120* mutant (Fig 7A). Fourth, like *trs120* mutant during cytokinesis, *ypt3-i5* cells accumulated vesicles (arrowheads) and tubulovesicular structures (arrows) at the division site (Fig 7C and 7D; S6D Fig), with similar distance to the plasma membrane (S6D Fig, yellow arrows and arrowheads), suggesting that the events after tethering, likely the fusion step, was affected by *ypt3* mutation. Lastly, *trs120* mutations displayed synthetic genetic interactions with *ypt3-i5* mutation (S6E and S6F Fig). Collectively, our data indicate that the TRAPP-II complex works with Rab11 GTPase Ypt3 for membrane deposition along the cleavage furrow during cytokinesis.

### Ectopic Targeting of Secretory Vesicles and Tubulovesicular Membrane Structures to Mitochondria by the TRAPP-II Complex

**Fig 9:**
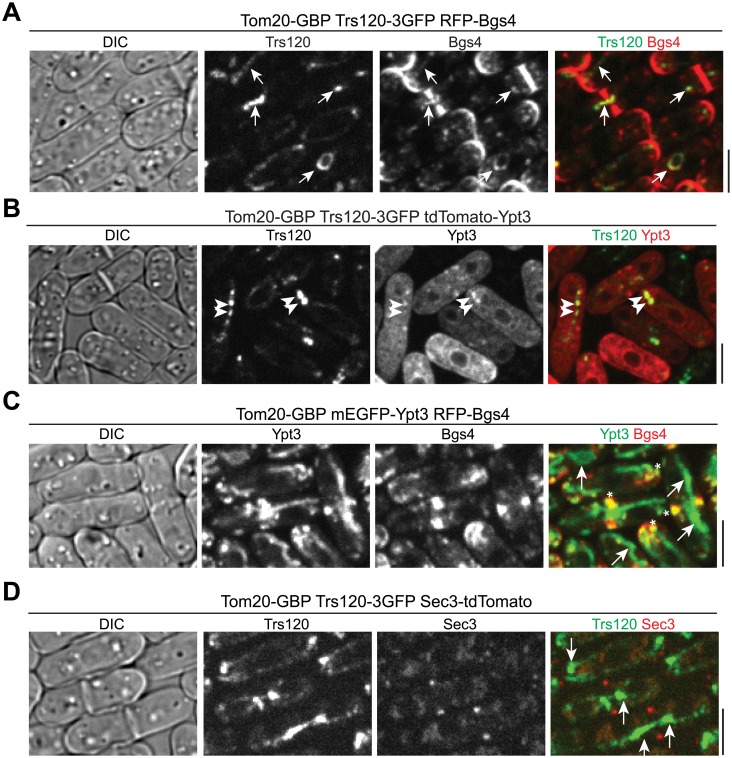
Mislocalized TRAPP-II complex but not Ypt3 can ectopically target vesicle cargos to mitochondria. (A) Micrographs of cells expressing Tom20-GBP Trs120-3GFP RFP-Bgs4. Colocalization of Trs120 with vesicle cargo Bgs4 on some mitochondrial structure is marked by arrows. (B) Mislocalization of Ypt3 to mitochondrial structure (arrowheads) by Trs120. (C) Mistargeted Ypt3 cannot recruit Bgs4 to mitochondria (arrows), although Ypt3 at its native location still colocalizes with Bgs4 at the division site and cell tips (asterisks). (D) Mistargeted Trs120 does not ectopically target the exocyst to mitochondria (arrows). Bars, 5 μm.

Although direct evidence from in vitro reconstitution that the exocyst tethers secretory vesicles to the plasma membrane is missing, it has been shown in budding yeast that ectopic targeting of Sec3 to mitochondria or peroxisomes results in the recruitment of secretory vesicles to these surrogate organelles [106]. To test whether TRAPP-II complex can target and/or tether Golgi-derived secretory vesicles/compartments, we mislocalized Trs120-3GFP to mitochondrial surface using the GFP-binding protein (GBP; [107]). We tagged the mitochondrial outer membrane protein Tom20 with GBP at its cytosol-exposed COOH-terminus [106]. GBP recognizes most GFP variants but not RFP or Tomato-tagged fluorescence proteins (S7A and S7B Fig; [107]). We used RFP or tdTomato tagged proteins to check whether other components of TRAPP-II complex or cargos of secretory vesicles/compartments are mistargeted by mislocalized Trs120-3GFP. Consistent with the reported central role of Trs120 in TRAPP-II assembly [108], mislocalized Trs120 could recruit other components of TRAPP-II complex, such as Trs130, to mitochondria (S7A Fig). Importantly, β-glucan synthase Bgs4 labeled secretory vesicles/compartments colocalized with Trs120 on mitochondria (arrows) in 75% of *tom20-GBP trs120-3GFP* cells (*n* = 324 cells; Fig 9A), suggesting that the TRAPP-II complex can recruit and possibly tether vesicles.

Consistent with our conclusion that the TRAPP-II complex works preferentially with the Rab11 GTPase Ypt3, we detected mitochondrial localization of Ypt3 in 80% of *tom20-GBP trs120-3GFP* cells (*n* = 211 cells; Fig 9B, arrowheads). In contrast, mislocalized Trs8502 (the TRAPP-III component Trs85) did not lead to mitochondrial localization of Ypt3 (S7C Fig, arrows). Ectopically targeted Ypt3 did not recruit Bgs4 to mitochondria (Fig 9C), suggesting that the Rab GTPase itself was not sufficient to tether vesicles. In addition, the exocyst was not mistargeted by ectopically localized TRAPP-II (Fig 9D), although TRAPP-II and exocyst complexes physically interact in plant cells [42]. Together, these data suggest that the TRAPP-II complex works together with Ypt3 to promote vesicles/compartments tethering, although whether TRAPP-II functions as a GEF to activate Ypt3-dependent tethering activities or as a direct tether itself is still unclear.

**Fig 10:**
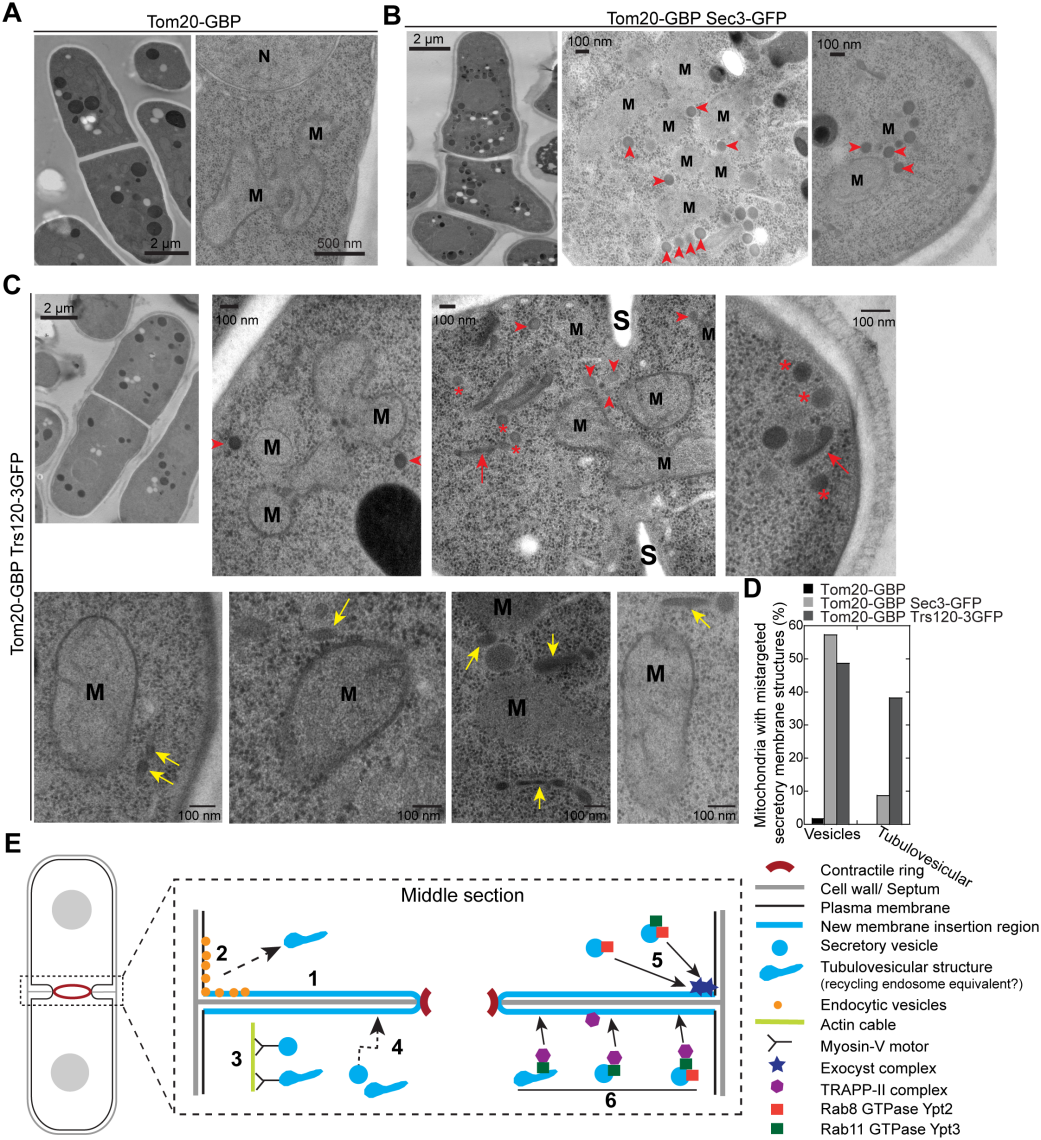
Mislocalized TRAPP-II complex can ectopically target vesicles and tubulovesicular membrane structures to mitochondria. (A–D) EM images (A–C) and quantification (D) showing post-Golgi secretory vesicles (arrowheads) or elongated tubulovesicular membrane structures (yellow arrows, maybe specialized late-Golgi cisternae or recycling endosomes) mis-targeted and tethered to mitochondria (M) in cells expressing Tom20-GBP Sec3-GFP (B) or Tom20-GBP Trs120-3GFP (C) but not Tom20-GBP alone (A). Some free vesicles (asterisks) or tubulovesicular structures (red arrows) accumulated in the cytoplasm or near the active growth sites were marked. N = Nucleus, S = septum. (D) Quantification of the mitochondria associated with mis-targeted secretory vesicles or tubulovesicular structures. (E) A working model for the roles of the exocyst and TRAPP-II complexes in vesicle trafficking and membrane deposition at the cleavage furrow during cytokinesis. The events 1–6 are symmetrical at the division site and omitted at some locations for clarity. 1. New membrane is deposited throughout the cleavage furrow. 2. Endocytic vesicles are mostly generated at the rim of the division plane or the adjacent regions and may become tubulovesicular structures like recycling endosomes. 3. Myosin-Vs transport secretory vesicles or recycling endosome equivalents along actin cables to the division plane. 4. Secretory vesicles can also reach the division site by actin-independent random walk. 5. Exocyst complexes localize to the rim of the cleavage furrow and preferentially tether 90-nm secretory vesicles probably through interaction with Rab8 GTPase Ypt2. 6. TRAPP-II complexes localize along the cleavage furrow (slightly biased to the leading edge) to directly tether or indirectly promote the tethering of Rab11 GTPase Ypt3-labeled recycling endosome equivalents or 90-nm secretory vesicles.

To confirm that secretory vesicles/compartments are tethered to mitochondria in *tom20-GBP trs120-3GFP* cells, EM was performed as described previously [89]. In cells expressing Tom20-GBP only, vesicles were not detectable at mitochondria (distance of vesicles to mitochondria <100 nm) in EM thin sections (*n* = 12 cells; Fig 10A and 10D). As a positive control, in cells expressing Tom20-GBP Sec3-GFP, we observed attachment of secretory vesicles with a diameter of ~90 nm to ~60% mitochondria from all imaged cells with 3.0 ± 0.9 vesicles per mitochondria (*n* = 12 cells; Fig 10B and 10D, arrowheads). Similar to the budding yeast strain carrying comparable constructs [106], mitochondria were clustered in this mutant (Fig 10B). Similar secretory vesicles were also detected to associate with mitochondria in ~70% cells expressing Tom20-GBP Trs120-3GFP (*n* = 14 cells; Fig 10C, arrowheads), with ~1.5 ± 0.4 vesicles per mitochondria detected in ~50% mitochondria (Fig 10C and 10D). In addition, ~40% of mitochondria had attached tubulovesicular membrane structures (Fig 10C, yellow arrows; Fig 10D), suggesting that secretory compartments resembling recycling endosomes were also mistargeted. However, only ~9% of mitochondria in t*om20-GBP sec3-GFP* cells attached such structures (Fig 10D), indicating exocyst complex prefers to tether spherical vesicles. In addition, some vesicles (Fig 10C, asterisks) and tubulovesicular structures (Fig 10C, red arrows) were either free in the cytoplasm or accumulated at the active growth site, possibly caused by the depletion of TRAPP-II from its native localizations. Together, secretory vesicles and the tubulovesicular structures can be retargeted and tethered to mitochondria by mislocalized TRAPP-II complex. Together, our data indicate that the TRAPP-II complex is critical for tethering Ypt3-containing vesicles or other secretory compartments to the plasma membrane.

## Discussion

Previous studies found that new plasma membrane was predominantly inserted at the leading edge of the cleavage furrow during cytokinesis in animal cells [7,25–27]. In this study, we challenged the universality of the model with our finding that post-Golgi secretory vesicles and compartments are delivered to everywhere along the cleavage furrow for membrane insertion during fission yeast cytokinesis (Figs 2 and 3). The involvement of exocyst complex as a vesicle tether in cytokinesis was confirmed (Fig 10B). However, the fact that the exocyst localizes to the rim of the division plane but vesicle fusion and membrane insertion do not happen exclusively at the rim indicates that other vesicle tether or tethering factor is involved. Indeed, we find that the putative vesicle tether TRAPP-II complex localizes to the cleavage furrow and is also involved in vesicle tethering and membrane insertion during cytokinesis (Figs 5–10; see the proposed model in Fig 10E).

### Where Is the New Membrane Inserted during Furrow Ingression in Cytokinesis?

In many animal cells, the whole exocytosis machinery including the microtubule-dependent vesicle transport, the tethering of vesicles by exocyst complex, and many membrane trafficking regulators is targeted preferentially to the leading edge of (or beneath) the cleavage furrow during cytokinesis [7]. For examples, post cellularized *Drosophila* embryos target both F-actin and membrane as a unit to the leading edge of the invaginating furrows [25]. Ral GTPases RalA concentrates and stabilizes the exocyst at the leading edge as rings during furrow ingression in early cytokinesis [109]. Loncar and Singer (1995) showed that vesicles line up and fuse ahead of the cleavage furrow in EM [110]. Thus, the prevailing model is that new membrane is inserted at the cleavage furrow tip. Given that membrane expansion can be a separate process both temporally and spatially from ring constriction in some animal cells [5], it might be enough to target just a small amount of new membrane but mainly cytokinesis proteins through vesicle trafficking to the leading edge of cleavage furrow during cytokinesis. However, significant membrane expansion and furrow invagination appears inseparable in fission yeast and many other cell types [6,111]. It might be more efficient to insert new membrane along the whole cleavage furrow instead of just to the furrow tip. Spreading vesicle insertion sites may avoid crowding of vesicles and the vesicle fusion machineries, especially in cell types that require a large amount of new membrane during cytokinesis. Consistently, new membrane addition in a broader region along the cleavage furrow have been detected in *Xenopus* embryos and zebrafish blastomeres [19,112].

Our discovery that new membrane from secretory vesicles/compartments is not predominantly inserted adjacent to the contractile ring raises an interesting question on the coordination between membrane deposition and ring constriction during cytokinesis. It has been proposed that the contractile ring recruits secretory compartments to the division site and the F-BAR protein Cdc15 in the ring helps to deliver Bgs1, a vesicle cargo, to the cleavage furrow [55, 87]. Our data are not incompatible with that proposal, although these pioneer studies do not have enough spatiotemporal resolutions on the exocytic events. Cdc15 may mainly help recruit Bgs1 to the division site during ring maturation when they colocalize but not during ring constriction. Ring constriction may help to generate more vesicle insertion sites at the cleavage furrow. The distribution of the formin For3 and myosin-V motor Myo52 at the division site (Fig 3) is consistent with this possibility. Alternatively, ring constriction may generate tension on the existing plasma membrane in the cleavage furrow and this tension guides membrane expansion by facilitating exocytosis and inhibiting endocytosis. Similar hypotheses or models have been proposed for membrane translocation during cell motility [113,114]. It will be interesting to test these possibilities in future studies.

### The Function and Coordination of the Exocyst and TRAPP-II Complexes during Cytokinesis

The exocyst complex is the best known presumptive vesicle tether at the plasma membrane during cell polarization, cell motility, cytokinesis, and many other cellular processes [30,31,115]. However, direct evidence of exocyst’s vesicle tethering activity from in vitro reconstitution is missing [31]. We found that fission yeast exocyst can be mistargeted to mitochondria by its component Sec3 and then recruits post-Golgi secretory vesicles to mitochondria (Fig 10B), which strongly supports its vesicle tethering ability in *S*. *pombe*.

The TRAPP complex was initially indicated as a tethering factor for ER-derived vesicles in budding yeast [116]. The TRAPP-I complex plays an essential role in ER-to-Golgi transport [117]. TRAPP-II is proposed to affect intra- and post-Golgi traffic [37,39,40]. TRAPP-III is for autophagosome formation [37]. Like the exocyst, vesicle tethering ability and specificity of the TRAPP complexes have not been reconstituted in vitro [118]. Instead, their potential functions as the GEFs for Rab/Ypt GTPases have been studied in several different systems [37,94,95]. Although our data did not unambiguously assign TRAPP-II as a genuine MTC, they do indicate that TRAPP-II can recognize some secretory vesicles/compartment and promote vesicle tethering and fusion to the plasma membrane at the division site during cytokinesis.

Given the different localizations of the TRAPP-II and exocyst complexes at the division site, we favor a model that the TRAPP-II and the exocyst function at different locations, although both regulate membrane addition and dynamics at the division site (Fig 10E). This model is further supported by the observation that localization of the glucan synthase Bgs1 and glucanase Eng1 was affected differently in *trs120* and *sec8* mutants (Fig 7E–7G). Our data suggest that TRAPP-II may preferentially tether (or help to tether) Ypt3-labeled secretory vesicles as well as the tubulovesicular structures, whose identity is still unclear at this moment, along the cleavage furrow (Fig 10E). It is of great interest to examine the contribution of TRAPP-II’s potential GEF activity to vesicle tethering in the future. In contrast, it appears that the exocyst prefers to tether the typical spherical secretory vesicles together with Rab8 GTPase Ypt2 at the rim of the division plane (Fig 10E), given that the exocyst is an effector of the Rab8 GTPase Sec4 in budding yeast [119], which primarily locates on the surface of the secretory vesicles [120]. Because a portion of exocyst complexes are transported to the cell tips together with secretory vesicles by random walk [56], therefore we cannot rule out that a small fraction of exocyst may contribute to vesicle tether along the cleavage furrow. Exocyst and TRAPP-II may have overlapping localization during ring maturation given that they both start to concentrate at the cell equator during this stage (Figs 1G and 5A; S6 Video). However, the localization independence between the two complexes (S4E–S4G Fig) and the lack of mis-targeting of Sec3 by Trs120 (Fig 9D) does not support any physical interactions between them, in contrast to plant cells [42]. Therefore, TRAPP-II and exocyst complexes are both involved but might function independently at the division site to regulate the tethering of secretory vesicles/compartments for delivering cargos and inserting new membrane.

The role of endocytosis during cytokinesis is intriguing [6,121]. It was suggested that the recycling endosomes derived from endocytosis at other locations provide new membrane to the ingressing cleavage furrow [6,7,122]. However, this does not explain the role of endocytic events at the division site. Our finding that the rim of the division plane is the dominant sites for endocytosis (Fig 4) suggests that endocytosis can help to redistribute the plasma membrane and cytokinesis proteins from the rim (tethered by the exocyst) to the interior of the cleavage furrow (tethered by the TRAPP-II complex and/or other proteins). Thus, endocytosis may not only be important for retrieving and recycling extra plasma membrane but also actively contribute to membrane expansion at the division site during cytokinesis (Fig 10E).

In conclusion, we found an extensive addition of new membrane along the cleavage furrow rather than exclusively near the contractile ring or the rim of the division plane during cytokinesis. Our investigation of the TRAPP-II complex as another potential vesicle tether (directly or indirectly through Ypt3) besides the exocyst in cytokinesis opens up avenues to understanding the coordination of multiple vesicle tethering factors for a single task.

## Materials and Methods

### Strain Constructions, Genetic and Cellular Methods

S1 Table lists the *S*. *pombe* strains used in this study. We used standard genetic and PCR-based gene targeting methods to transform yeast cells and construct strains [123,124]. Tagged *trs120*, *trs130*, *trs8502*, *syb1*, *sec3*, *fim1*, *for3*, *myo52*, *sec72*, *rlc1*, and *tom20* genes are under the control of their endogenous promoters and integrated at their native chromosomal loci to replace the native genes. The N-terminal tagged *psy1*, *bgs1*, and *bgs4* strains (derived from gift strains from other labs) have a copy of tagged gene under the control of the endogenous promoters inserted in *leu1* locus with the native genes deleted (see S1 Table).

The N-terminal tagging of *ypt3* and *ypt2* was made by first cloning the promoters of *ypt3* (-971 to +6 bp) and *ypt2* (-882 to +6 bp) into the pFA6a-kanMX6-P3nmt1-mEGFP or pFA6a-kanMX6-P3nmt1-tdTomato vector at *Bgl*II and *Pac*I sites to replace the *3nmt1* promoter. The resulting plasmids (JQW896 for *mEGFP-ypt3*, JQW898 for *tdTomato-ypt3*, and JQW913 for *mEGFP-ypt2*) were used as templates to amplify *kanMX6-Pypt3-mEGFP*, *kanMX6-Pypt3-tdTomato*, and *kanMX6-Pypt2-mEGFP* fragments flanked with homologous sequences corresponding to the last 70 bp of 5′ untranslated region (UTR) and the complement of the first 70 bp of the coding sequence of *ypt3* or *ypt2*. The amplified and column purified PCR products were then transformed into wt strain JW81 as described [124]. The positive transformants from visual screen were confirmed by PCR. Plasmid for expression of Ypt3 (pSM925, pREP41-tdTomato-ypt3, a gift from Sophie Martin) was under the control of inducible *41nmt1* promoter [72]. The plasmid was transformed into yeast using standard method [124]. The cells were cultured in Edinburgh minimal medium plus five supplements (EMM5S) without leucine (EMM5S –leucine) for 20–24 h to induce the expression of *41nmt1* promoter before imaging.

To generate *trs120* point mutants, we used the marker reconstitution mutagenesis method as previously described [89]. Briefly, *trs120* gene including 70 bp of 5′ UTR, the open reading frame (ORF), introns, and 137 bp of 3′ UTR was amplified from genomic DNA and cloned into a plasmid with the C-terminus of *his5* ORF to obtain the plasmid JQW886 (*trs120*-*his5* ^*c*^). In addition, we inserted *his5* ORF (without its C-terminus) immediately after 3′UTR of *trs120* locus to generate strain JW6842. Error-prone PCR was performed to amplify *trs120* fragment from JQW886, which was then transformed into JW6842 strain. We selected *trs120* mutants with EMM5S –histidine medium and checked their growth and morphology at different temperatures. ~150 temperature-sensitive mutants with strong cytokinesis-related phenotypes were selected. *trs120* sequences (including 5′ UTR, ORF, introns, and 3′ UTR) of 15 mutants were cloned and sequenced to identify the mutations, and the two least mutated strains (*trs120-ts1* and *trs120-M1*) were used for further experiments. *trs120-ts1* contained five missense mutations (I370T, L543P, T700A, K993I, and F1158V). *trs120-M1* contained one mutation in the coding sequence (L1113R), one mutation in the second intron (an A to T switch at the 24th bp of the intron), and one mutation in the 3′ UTR (84 bp downstream of the stop codon, T to C).

To test the functionalities of tagged Trs120, Trs130, Tom20, Ypt2, and Ypt3, the growth and morphology of the strains expressing tagged proteins were examined at different temperatures. Most strains resembled wt cells, indicating these tagged proteins are functional under the tested conditions. However, the *Pypt2-mEGFP-ypt2* strain displayed temperature-sensitive growth defects with increased septation index at 36°C. Thus, the strain was only used at 25°C. Although *trs120-ts1 ypt3-i5* was synthetic lethal, *trs120-ts1 Pypt3-mEGFP-ypt3* strain resembled *trs120-ts1*. Thus, mEGFP-Ypt3 is functional. *Pypt3-tdTomato-ypt3* cells formed Ypt3 containing aggregates in the cytoplasm (not detected in *Pypt3-mEGFP-ypt3* strain), stopped growing at 36°C, and displayed synthetic lethal interactions with other mutations, indicating tdTomato-Ypt3 is not functional. Therefore, we used the plasmid-born tdTomato-Ypt3 (pSM925) when necessary.

All drug treatments were performed in micro-centrifuge tubes at 25°C and then cells were imaged on bare slides to maintain the drug concentration except where noted. BFA (Sigma, B7651) treatment was performed at a final concentration of 50 μg/ml from a 5 mg/ml stock solution in ethanol for 10 min with rotation [125]. To test whether cortical localization of Syb1 at the division site depends on microtubules, cells were treated with MBC at a final concentration of 25 μg/ml for 10 min and then imaged on gelatin slide with the same drug concentration [126].

### Acid Phosphatase Secretion Assay

Acid phosphatase secretion was measured as follows (modified from [33]). Cells were grown to log phase in YE5S rich medium at 25°C, pelleted, washed twice with equal volume EMM5S medium, and resuspended in fresh EMM5S and shifted to 36°C at time 0. Samples were taken each hour. The absorbance at 595 nm was measured for cell concentrations. Then 1 ml cells for each culture was centrifuged, and 500 μl of the supernatant was added to 500 μl of substrate solution (2 mM *p*-nitrophenyl phosphate, 0.1 M sodium acetate, pH 4.0; prewarmed to 30°C) and incubated at 30°C for 5 min. Reactions were stopped by the addition of 500 μl of 1 M sodium hydroxide. The absorbance of the reaction solution at 405 nm was measured using the substrate solution with 500 μl of EMM5S as a blank. The OD_405_/OD_595_ value versus time was plotted.

### Microscopy and FRAP

Microscopy was performed as previously described [127]. Briefly, cells were grown exponentially in liquid YE5S medium at 25°C for 36–48 h before microscopy at 23–24°C except where noted. Cells were collected by centrifugation at 3,000 rpm for 30 s and washed twice with EMM5S medium to reduce autofluorescence for fluorescence microscopy. Live-cell fluorescence microscopy at 23–24°C was performed using a thin layer of EMM5S liquid medium with 20% gelatin (Sigma-Aldrich) and 5 μM n-propyl-gallate (n-PG). For fluorescence microscopy at 36°C, cells were first grown at 25°C for 36 h, then shifted to 36°C for a given time (see Fig legends). For imaging preparation, cells grown at 36°C were washed and concentrated in pre-warmed YE5S liquid medium with 5 μM n-PG. Then 2-μl of the concentrated cells were spotted onto a coverglass-bottom dish (Delta TPG Dish; Biotechs, Butler, PA, United States), covered with the pre-warmed YE5S agar, and imaged at 36°C in a preheated climate chamber (stage top incubator INUB-PPZI2-F1 equipped with UNIV2-D35 dish holder; Tokai Hit, Shizuoka-ken, Japan).

Tetrad fluorescence microscopy for *trs120* ^+^ */trs120*Δ diploid (Fig 6A and 6B) was performed as previously described at 23–24°C [77] with the following modifications: we started the ~14 h of imaging after 20–24 h of cell growth following tetrad dissection at 25°C. To minimize phototoxicity, Rlc1 channel was imaged every 5 min with low laser power (5%–7.5%).

Nikon 100×/1.4 NA Plan-Apo objective lenses were used for all imaging. The spinning disk confocal system (UltraVIEW Vox CSUX1 system, Perkin Elmer Life and Analytical Sciences) with 440-, 488-, 515-, and 561-nm lasers and back-thinned EMCCD camera (Hamamatsu C9100-13) on a Nikon (Nikon, Melville, NY, US) Ti-E microscope was used to collect fluorescence images for all figures and videos except Figs 5A, 7E and 8B. The pixel size of the images under our imaging conditions is 144 nm/pixel. Since we used the location of the brightest pixel to represent the center of a specific vesicle/endosome, the resolution in the *x*-*y* direction is ~150 nm. For Figs 5A, 7E and 8B, another spinning disk confocal system (UltraVIEW ERS; PerkinElmer) with 440- and 568-nm solid state lasers and 488- and 514-nm argon ion lasers, and a cooled charge coupled (CCD) device camera (ORCA-AG; Hamamatsu Photonics) on a Nikon microscope (Eclipse TE2000-U) was used with 2 × 2 binning. Under our imaging conditions, no bleed-through between green and red channels was detected.

To track vesicle movement, the middle focal plane of cells was imaged with a speed of 2–5 frames per second (fps) for the vesicle channel in 2- or 3-min movies. The Rlc1 channel was imaged once every minute. DIC images were taken as snapshots immediately before and after the fluorescence movies to make sure that there was no focal shifting. To reduce the interference from Syb1 or Ypt3 signals at the division site on vesicle tracking, we carried out FRAP to bleach the Syb1 or Ypt3 signals at the central ~1/4 to 1/3 of the whole cell using the photokinesis unit on the UltraVIEW Vox confocal system as described [127]. Then cells were imaged the same way as unbleached cells. Psy1 signals on the plasma membrane was also bleached in the same way. Briefly, the Psy1 signals on the plasma membrane of the division site or cell side were bleached at the selected ROI and cells were imaged for 20 min with 10-s interval to track recovery of the Psy1 signals.

To show the synthetic interactions between mutations with DIC images, a Nikon Eclipse Ti inverted microscope equipped with a Nikon cooled digital camera DS-Ql1 was used as before [128].

### Data Analysis and Vesicle Tracking

We analyzed images using ImageJ (National Institutes of Health), UltraVIEW (PerkinElmer), and Volocity (PerkinElmer). Fluorescence images in figures are maximum-intensity projections of z sections spaced at 0.4–0.5 μm except where noted. Movements of individual vesicles in movies were tracked using the plug-in MTrackJ in ImageJ [127,129]. The center positions (absolute values along the *x*- and *y*-axis in un-rotated images) of the vesicles were determined using the pixel with the highest fluorescence intensity. The last tractable position at or near the division plane from a continuous movement of an individual vesicle was recorded as the docking/deposition site of the specific vesicle. The diameters and positions of the contractile rings were determined by the brightest pixel of Rlc1 images on the middle focal plane using the first Rlc1 image at the beginning of movies. The outer boundaries (or rims) of cell-division planes were determined using the DIC images taken before the fluorescence movies. Since the division planes of the cells were random in the imaging field, their contractile-rings have an angle to the horizontal *x*-axis in the un-rotated images. The docking points of all tractable vesicles from an individual cell were rotated based on the angle between the contractile-ring/division plane and the *x*-axis using MATLAB software. The new *x* and *y* values of the docking sites, the positions and diameters of the rings, and the rims of the division plane were generated, but their relative positions were unchanged with the new *x*-axis marking the division plane. To plot data from multiple cells in one figure, we normalized the width of the division plane to 3.5 μm (the range of the values is from 3.4 to 3.9 μm). Given that the contractile ring was not always at the center of the division plane, the plot in Fig 2F was also normalized according to the center of the contractile-rings.

To analyze the assembly sites of endocytic patches/vesicles, Fim1-mEGFP images acquired at the middle focal plane with 4.14 fps in 2-min movies were used. The following criteria were used to identify newly assembled Fim1 patches from those internalizing endocytic vesicles that already pinched off from the plasma membrane [81,82]: (1) Fim1 signal gradually increases to reach a peak and (2) the patch does not move before reaching the signal peak. If these criteria were fulfilled, the location of the brightest pixel of a Fim1 patch when the peak signal reached was recorded as the endocytic vesicle assembly site, and the locations of these assembly sites were rotated and aligned as the secretory vesicles.

To quantify Psy1 recovery on the plasma membrane after photobleaching at the cell side of interphase cells, the rim of the division plane, or the leading edge of the cleavage furrow that is immediately behind the contractile-ring, a 9-pixel square was chosen within the bleached regions. Same-sized squares were used to measure cytoplasmic signal outside the bleached region over time as the background, which was deducted from the plasma-membrane intensity.

### Electron Microscopy

EM was done at the Boulder Electron Microscopy Services at University of Colorado, Boulder as described previously [89]. To detect accumulation of secretory vesicle or other secretory compartments in wt (JW81), *sec8*, *trs120*, and *ypt3* mutants, cells were grown exponentially at 25°C for 2 d, and then shifted to 36°C (except *ypt3* cells) for 2 or 4 h before harvesting at 36°C. To observe vesicles or tubulovesicular structures tethered to mitochondria in *tom20-GBP trs120-3GFP* and control strains, cells were grown at 25°C for 2 d and harvested at 25°C. Cells were prepared for EM as described previously [130]. Briefly, cells were harvested using Millipore filters. Specimen preparation includes high pressure freezing with the Wohlwend Compact 02 Freezer, freeze-substitution in the presence of 2% osmium tetroxide and 0.1% uranyl acetate in acetone. Cells were embedded in Epon-Araldite epoxy resin, and serially sectioned with a thickness of 70 nm. The samples were then post stained with uranyl acetate and lead citrate and observed on a Philips CM100 transmission electron microscope (FEI, Hillsboro, OR).

## Supporting Information

S1 Data Raw data for analyses and quantifications shown in the figures and supplemental figures, as indicated. (XLSX)

S1 Fig Membrane invagination and ring constriction are defective in exocyst mutant, and v-SNARE Syb1 and β-glucan synthase Bgs1 travel to the division site during ring maturation. (A, B) Membrane invagination and ring constriction are slower in exocyst mutant *sec8-1* even at the permissive temperature 25°C. (A) Micrograph of *sec8-1 GFP-Psy1 Rlc1-tdTomato* cells at the middle focal plane. (B) Time courses of membrane invagination and ring constriction in wt (top) and two *sec8-1* cells (bottom) as marked in (A). (C, D) Vesicles (arrowheads) containing Syb1 (C) or Bgs1 (D) are delivered to the division site during ring maturation. Bars, 5 μm.
(EPS)

S2 Fig EM images of wt cells during septum formation or maturation, tracking delivery of Syb1-labeled vesicles/compartments and Psy1 dynamics at the division site during septum maturation. (A) EM images of wt cells during septum formation or maturation. Secretory vesicles are marked by arrows. Elongated or irregular-shaped tubulovesicular structures are marked by arrowheads. Bars, 100 nm. (B, C) Time courses of Syb1 delivery (B) and distribution of its docking sites during septum maturation (C). Syb1 signal within the box at the division site was bleached at time 0 and incoming Syb1 vesicles/compartments in the middle focal plane were tracked in cells without Rlc1 signal but a full septum. Cells were imaged for 3 min after photobleaching. (B) Arrowheads mark travelling vesicles. (C) Quantification of the docking sites of all tractable Syb1 vesicles in the 3-min movies. The septum is positioned at *y* = 0. (D) Recovery of Psy1 signal on the plasma membrane at the division site (arrows) after bleaching at the region marked by red box. An interphase cell on the right was bleached and imaged as a control, which showed almost no recovery (arrowhead). Bars in B and D, 5 μm.
(EPS)

S3 Fig Cytokinesis defects in *myo52*Δ and tracking the movement of Myo52 puncta during different stages of cell division. (A, B) Montage (A) and quantification (B) showing the delay in ring constriction and cell separation in *myo52*Δ cells at 25°C. *, *p* < 0.01 compared with wt control. (C) Time courses showing Myo52 puncta moving to the division site during ring maturation, ring constriction, and septum maturation on middle focal plane. Moving Myo52 puncta are marked by arrowheads. (D) Distribution of the final destination of Myo52 puncta at the division site relative to the position of constricting ring from 5 cells (color coded) in 2-min movies. *X*- and *y*-axes are along the division site and cell long axis, respectively. The ring (the diameter marked by the color lines) is displaced along the *y*-axis away from its real position at *y* = 0 for clarity. (E) Distribution of the final destination of Myo52 puncta relative to the position of septum (at *y* = 0) during septum maturation in 2-min movies. Bars, 5 μm.
(EPS)

S4 Fig Synthetic genetic interactions between *trs120* and exocyst mutations, their independency for localization, and defective acid phosphatase secretion in the mutants. (A-C) Localization and dynamics of Trs120. (A, B) Sum projections of Trs120-3GFP from the single focal plane in 2-min movies along with DIC and Rlc1-tdTomato images taken at time 0. (B) Right: kymograph showing Trs120 at the division site (marked by the blue line on the duplicated sum image) during the 2-min movie. (C) Quantification the duration of Trs120 puncta staying on or close to the plasma membrane after they arrive at the division site as illustrated by the paired red lines in (B). (D) Growth of wt, *trs120-ts1*, *sec8-1*, and *trs120-ts1 sec8-1* cells on YE5S + PB medium at the 30°C. (E) Trs120 localization at the division site (arrowheads) in wt and *sec8-1* cells at 25°C. (F-G) Sec3 localization in wt and *trs120* mutants at 25°C (F) or 36°C (G, grown at 36°C for 2 h and imaged at 36°C). (H) Acid phosphatase secretion assay for wt, *trs120*, and *sec8* mutants grown at 36°C (see Materials and Methods). Bars, 5 μm.
(EPS)

S5 Fig Ypt3 labeled secretory vesicles/compartments travel to the division site during cytokinesis. (A) Trs120 and Ypt2 only colocalize (arrowheads) on a small fraction of punctate structures close to the division site or cell tips. Enlarged images of the middle focal plane of the boxed regions are shown on the right. (B) Ypt3 vesicles/puncta travel with (left, boxes) or without Ypt2 (right, arrows). (C) Images (left) and quantification (right, *n* = 136 puncta) showing the partial colocalization of Ypt3 and Bgs4 (arrowheads). (D) Two examples showing the different dynamics of Ypt3 puncta at the division site of a cell with a constricted ring after photobleaching. The white dashed rectangle marks the bleached region. Yellow box labels a punctum docking/tethering to the center of the division plane for 7 s before the signal spreads out/disappears. Red box marks a punctum that stays for >20 s at the rim of the division site before the signal spreads out/disappears. (E) Plasmid-borne tdTomato-Ypt3 labeled vesicle/compartments (arrows) travel to the division site during ring maturation. Bars, 5 μm.
(EPS)

S6 Fig Localization dependency of Ypt3 on the TRAPP-II complex, accumulation of secretory vesicles/compartments in *ypt3-i5* mutant, and genetic interactions between *ypt3* and *trs120* mutations. (A) Ypt3 cannot concentrate at the division site and cell tips in *trs120-ts1* mutant grown at 36°C for 2 h although the global protein levels are not affected (right). For quantification, the *mEGFP-ypt3* strains (in wt or *trs120-ts1*, outlined with yellow broken lines) are mixed with JW81 wt that has no fluorescence marker (red broken lines). (B) Localization of Trs120 in wt and *ypt3-i5* cells at 25°C. Red and yellow arrow indicates Trs120 accumulation at the division site and the cell tips, respectively. (C) Syb1 accumulates (arrows) at the division site or cell tips in *ypt3-i5* mutant grown in YE5S medium at 36°C for 2 h. (D) EM images showing the accumulation of secretory vesicles (arrowheads) and elongated tubulovesicular membrane structures (arrows) in *ypt3-i5* mutant grown at 25°C. Yellow arrowheads and arrows mark the vesicles or tubulovesicular structures are within 100 nm to the plasma membrane. (E) The predicted *trs120-M1 ypt3-i5* double mutant (in red circles) is inviable on YE5S plate at 25°C after tetrad dissection. (F) The growth of wt, *trs120-ts1*, *ypt3-i5*, and *trs120-ts1 ypt3-i5* cells on YE5S + PB medium at 30°C. The double mutant does not grow at this semi-permissive temperature for single mutants. Bars, 5 μm.
(EPS)

S7 Fig Ectopically targeted Trs120 can recruit other component of TRAPP-II complex and controls for ectopic targeting experiments. (A) Mitochondrial targeted Trs120 by Tom20-GBP can recruit TRAPP-II component Trs130 to mitochondria (top panels). Bottom panels show controls that Tom20-GBP does not directly recruit tdTomato tagged Trs130 to mitochondria and no signal bleed through between red/green channels when *tom20-GBP trs120-3GFP* cells and *tom20-GBP trs130-tdTomato* cells were mixed and imaged using the same imaging settings as those in top panels. (B) Tom20-GBP does not directly recruit RFP tagged Bgs4 to mitochondria and no signal bleed through between red/green channels when *tom20-GBP trs120-3GFP* cells and *tom20-GBP RFP-bgs4* cells were mixed and imaged using the same imaging settings as those in Fig 9A. (C) Mis-targeted Trs8502 (arrows, putative TRAPP-III component) cannot recruit Ypt3 to mitochondria. Bars, 5 μm.
(EPS)

S1 Table *S*. *pombe* strains used in this study. (DOCX)

S1 Video Movement of GFP-Syb1 vesicles to the division site. Three stages of cytokinesis at the beginning of the time-lapse movies (contractile-ring maturation, constriction, and septum maturation) are marked with Rlc1-tdTomato and shown in the lower panels. The movies focus on the middle focal plane of the cell and images were taken at a rate of 3 fps for the Syb1 channel for 1 min. This video corresponds to Fig 2B and 2C. Display rate: 6 fps.
(MOV)

S2 Video Movement of GFP-Syb1 vesicles observed after bleaching Syb1 signal at the division site. The division site signal of Syb1 within the region of interests (ROIs) was bleached at the middle focal plane in two cells with constricting rings, and then cells were imaged at 0.5 s interval for 2 min. Images of DIC, Rlc1-tdTomato, and GFP-Syb1 taken immediately before bleaching are shown at the beginning of the movie. Bleached regions are marked by red boxes on GFP-Syb1 image taken before bleaching. This video corresponds to Figs 2D–2F and S2B. Display rate: 6 fps after bleaching.
(MOV)

S3 Video Dynamics of GFP-Psy1 at the division site during cytokinesis. Psy1 signal within the ROIs was bleached at cell sides in interphase cells (top) or at the division site in the cell with a constricting ring (bottom) at the middle focal plane and imaged at 10-s interval for 20 min. DIC and Rlc1-tdTomato images were taken every minute and shown in the left and middle panels. This video corresponds to Figs 2I–2K and S2D. Display rate: 6 fps.
(MOV)

S4 Video Movement of Myo52-GFP puncta to the division site during three stages (as in S1 Video) of cytokinesis. The contractile-ring is marked with Rlc1-tdTomato and imaged once every minute (shown in the lower panels). Myo52 was imaged with 0.5-s interval for 3 min. This video corresponds to S3C–S3E Fig. Display rate: 6 fps.
(MOV)

S5 Video Tracking of endocytic events using Fim1-mEGFP. The movie focused at the middle focal plane of the cells and the interval for Fim1 images was 0.24 s. The ring (middle panels and red in the bottom panels) is marked with Rlc1-tdTomato and was imaged once every minute. Two fields including cells at different stages of cytokinesis are shown. This video corresponds to Fig 4. Display rate: 8 fps.
(ZIP)

S6 Video Trs120-3GFP puncta dynamically localize to the division site after ring assembly and last until cell separation. The cytokinesis nodes and the ring are labeled with Rlc1-tdTomato (middle panel). The maximum intensity projections of Rlc1 and Trs120 images from 13 slices spaced at 0.5 μm at each time point are shown. The length of the movie is 80 min with 2-min interval. Display rate: 6 fps.
(MOV)

S7 Video Movement of Trs120-3GFP puncta to the division site during different stages of cytokinesis marked by Rlc1-tdTomato. The middle focal plane of the cells was imaged every 0.5 s for Trs120 and 30 s for Rlc1 for 2 min. Two fields including cells at different stages of cytokinesis are shown. This video corresponds to Fig 5G and 5H. Display rate: 6 fps.
(MOV)

S8 Video Movement of mEGFP-Ypt3 vesicles to the division site during different stages of cytokinesis marked by Rlc1-tdTomato. The middle focal plane of the cells was imaged every 0.5 s for Ypt3 (green) and 60 s for Rlc1 (red) for 2 min. Two fields including cells at different stages of cytokinesis are shown. Display rate: 6 fps.
(MOV)

S9 Video Movement of mEGFP-Ypt3 labeled vesicles/compartments observed after bleaching Ypt3 signal at the division site. The division site signal of Ypt3 within the boxed regions was bleached at the middle focal plane in three cells, and then cells were imaged at 0.5 s interval for 3 min. Images of DIC, Rlc1-tdTomato, and mEGFP-Ypt3 taken immediately before bleaching are shown at the beginning of the movie. This video corresponds to Figs 8D–8G and S5D. Display rate: 6 fps after bleaching.
(MOV)

## Abbreviations

BFA: Brefeldin A
DIC: differential-interference contrast
fps: frames per second
EM: electron microscopy
EMM5S: Edinburgh minimal medium plus five supplements
ER: endoplasmic reticulum
FRAP: fluorescence recovery after photobleaching
GBP: GFP-binding protein
GEF: guanine nucleotide exchange factor
MBC: methyl benzimidazole-2-yl carbamate
mEGFP: monomeric enhanced green fluorescent protein
MTC: Multisubunit tethering complex
PB: phloxin B
ROI: region of interest
SD: standard deviation
SEM: standard error of the mean
SNARE: Soluble *N*-ethylmaleimide-sensitive factor activating protein receptor
tdTomato: tandem dimer Tomato
TRAPP-II: transport protein particle II
UTR: untranslated region; wt, wild type
