# Supplementary material for: Roles of the TRAPP-II Complex and the Exocyst in Membrane Deposition during Fission Yeast Cytokinesis
Source: PLoS Biol. 2016 Apr 15;14(4):e1002437. doi: 10.1371/journal.pbio.1002437 (PMC4833314; doi:10.1371/journal.pbio.1002437)
Supplement: S1 Table — (DOCX) [file pbio.1002437.s009.docx]

**Supporting Information**

**S1 Table. *S. pombe* strains used in this study.**

| **Strain** | **Genotype** | **Figure/video /reference** |
| --- | --- | --- |
| JW2402 | *psy1Δ::kanMX6 leu1^+^::GFP-psy1 rlc1-tdTomato-natMX6 ade6-M210 ura4* | Fig 1; S1 Fig; [1] |
| JW6548 | *h^+^ GFP-syb1-kanMX6 rlc1-tdTomato-natMX6 ade6 leu1-32 ura4-D18* | Fig 1; S1 Fig |
| JW5249 | *GFP-bgs1-leu1^+^ bgs1Δ::ura4^+^ rlc1-tdTomato-natMX6 ade6-M210 leu1-32 ura4-D18* | Fig 1; S1 Fig |
| JW7062 | *sec3-GFP-kanMX6 rlc1-tdTomato-natMX6 ade6 leu1-32 ura4-D18* | Fig 1 |
| JW6967 | *sec8-1 psy1Δ::kanMX6 leu1^+^::GFP-psy1 rlc1-tdTomato-natMX6 ade6-M210 leu1-32 ura4* | S1 Fig |
| JW7062 | *sec3-GFP-kanMX6 rlc1-tdTomato-natMX6 ade6 leu1-32 ura4-D18* | Fig 2 |
| JW6548 | *h^+^ GFP-syb1-kanMX6 rlc1-tdTomato-natMX6 ade6 leu1-32 ura4-D18* | Fig 2; S2 Fig |
| JW81 | *h^-^ ade6-210 ura4-D18 leu1-32* | Fig 2; S2 Fig; [2] |
| MBY887 | *h^+^ sec8-1 leu1-32 ura4-D18* | Fig 2; [3] |
| JW2402 | *psy1Δ::kanMX6 leu1^+^::GFP-psy1 rlc1-tdTomato-natMX6 ade6-M210 ura4* | Fig 2; S2 Fig; [1] |
| JW6548 | *h^+^ GFP-syb1-kanMX6 rlc1-tdTomato-natMX6 ade6 leu1-32 ura4-D18* | Fig 3 |
| JW6965 | *for3Δ::kanMX6 GFP-syb1-kanMX6 rlc1-tdTomato-natMX6 ade6 leu1-32 ura4-D18* | Fig 3 |
| JW7002 | *myo52Δ::ura4^+^ GFP-syb1-kanMX6 rlc1-tdTomato-natMX6 ade6 leu1-32 ura4-D18* | Fig 3 |
| JW2292 | *kan^S^ Pmyo2-mCFP-myo2 for3-3YFP-kanMX6 ade6 leu1-32 ura4-D18* | Fig 3; [4] |
| JW6982 | *myo52-GFP-kanMX6 rlc1-tdTomato-natMX6 ade6 leu1-32 ura4-D18* | Fig 3; S3 Fig |
| JW5249 | *GFP-bgs1-leu1^+^ bgs1Δ::ura4^+^ rlc1-tdTomato-natMX6 ade6-M210 leu1-32*  *ura4-D18* | S3 Fig |
| JW7000 | *myo52Δ::ura4^+^ GFP-bgs1-leu1^+^ bgs1Δ::ura4^+^ rlc1-tdTomato-natMX6*  *ade6-M210 leu1-32 ura4-D18* | S3 Fig |
| JW6771 | *fim1-mEGFP-kanMX6 rlc1-tdTomato-natMX6 ade6 leu1-32 ura4-D18* | Fig 4 |
| JW7290 | *trs120-3YFP-kanMX6 sad1-mCFP-kanMX6 ade6-M210 leu1-32 ura4-D18* | Fig 5 |
| JW6726 | *anp1-GFP::ura4^+^ trs120-tdTomato-natMX6 ade6 leu1-32 ura4-D18* | Fig 5 |
| JW6729 | *sec72-GFP::ura4^+^ trs120-tdTomato-natMX6 ade6 leu1-32 ura4-D18* | Fig 5 |
| JW6732 | *bgs4∆::ura4^+^ Pbgs4^+^::GFP-bgs4^+^-leu1^+^ trs120-tdTomato-natMX6*  *ade6-M210 leu1-32 ura4-D18 his3-D1* | Fig 5 |
| JW6783 | *GFP-syb1-kanMX6 trs120-tdTomato-natMX6 ade6 leu1-32 ura4-D18* | Fig 5 |
| JW6705 | *h^-^ trs120-3YFP-kanMX6 ade6-210 leu1-32 ura4-D18* | Fig 5 |
| JW6724 | *for3Δ::kanMX6 trs120-3YFP-kanMX6 ade6 leu1-32 ura4-D18* | Fig 5 |
| JW6731 | *trs120-3GFP-kanMX6 rlc1-tdTomato-natMX6 ade6-M210 leu1-32*  *ura4-D18* | Fig 5; S4 Fig |
| JW3522 | *h^+^ rlc1-tdTomato-natMX6 ade6-M216 leu1-32 ura4-D18* | Fig 6^a^ |
| JW1341 | *h^-^ rlc1-tdTomato-natMX6 ade6-M210 leu1-32 ura4-D18* | Fig 6^a^; [5] |
| JW81 | *h^-^ ade6-210 ura4-D18 leu1-32* | Fig 6; S4 Fig; [2] |
| JW6842 | *h^-^ trs120-his5ΔC-kanMX6 his5Δ ade6-M210 leu1-32 ura4* | Fig 6 |
| JW7035 | *h^-^ trs120-M1-his5-kanMX6 his5Δ ade6-M210 leu1-32 ura4* | Fig 6; S4 Fig |
| JW7036 | *h^-^ trs120-ts1-his5-kanMX6 his5Δ ade6-M210 leu1-32 ura4* | Fig 6; S4 Fig |
| JW6713 | *h^-^ kanMX6-P81nmt1-mECitrine-trs120 ade6-210 leu1-32 ura4-D18* | Fig 6 |
| MBY887 | *h^+^ sec8-1 leu1-32 ura4-D18* | Fig 6; S4 Fig; [3] |
| JW7117 | *sec8-1 trs120-ts1-his5-kanMX6 his5Δ? ade6-M210 leu1-32 ura4* | Fig 6; S4 Fig |
| JW2716 | *h^+^ exo70Δ::kan^R^ ade6 leu1-32 ura4-D18* | Fig 6; [6] |
| JW7118 | *exo70Δ::kan^R^ trs120-ts1-his5-kanMX6 his5Δ? ade6 leu1-32 ura4* | Fig 6 |
| JW6742 | *sec3-tdTomato-hphMX6 trs120-3GFP-kanMX6 ade6-210 leu1-32* | S4 Fig |
| JW6704 | *h^-^ trs120-tdTomato-natMX6 ade6-210 leu1-32 ura4-D18* | S4 Fig |
| JW6862 | *sec8-1 trs120-tdTomato-natMX6 ade6-210 leu1-32 ura4-D18* | S4 Fig |
| IJ612 | *h^+^* *sec3-GFP-kanMX6 ade6-M216 leu1-32 ura4-D18* | S4 Fig; [7] |
| JW7114 | *trs120-M1-his5-kanMX6 his5Δ? sec3-GFP-kanMX6 ade6 leu1-32 ura4* | S4 Fig |
| JW7115 | *trs120-ts1-his5-kanMX6 his5Δ? sec3-GFP-kanMX6 ade6 leu1-32 ura4* | S4 Fig |
| JW6548 | *h^+^ GFP-syb1-kanMX6 rlc1-tdTomato-natMX6 ade6 leu1-32 ura4-D18* | Fig 7 |
| JW7173 | *trs120-ts1-his5-kanMX6 his5Δ? GFP-syb1-kanMX6 rlc1-tdTomato-natMX6*  *ade6 leu1-32 ura4* | Fig 7 |
| JW6867 | *sec8-1 GFP-syb1-kanMX6 rlc1-tdTomato-natMX6 ade6 leu1-32*  *ura4-D18* | Fig 7 |
| JW81 | *h^-^ ade6-210 ura4-D18 leu1-32* | Fig 7; [2] |
| JW7035 | *h^-^ trs120-M1-his5-kanMX6 his5Δ ade6-M210 leu1-32 ura4* | Fig 7 |
| PPG3723 | *h^-^  eng1-GFP-kan^R^ leu1-32 ura4-D18* | Fig 7; [8] |
| JW7311 | *trs120-M1-his5-kanMX6 eng1-GFP-kan^R^ leu1-32 ura4-D18* | Fig 7 |
| JW7310 | *sec8-1 eng1-GFP-kan^R^ leu1-32 ura4-D18* | Fig 7 |
| JW5249 | *GFP-bgs1-leu1^+^ bgs1Δ::ura4^+^ rlc1-tdTomato-natMX6 ade6-M210 leu1-32 ura4-D18* | Fig 7 |
| JW7294 | *sec8-1 GFP-bgs1-leu1+ bgs1Δ::ura4+ rlc1-tdTomato-natMX6 ade6-M210*  *leu1-32 ura4-D18* | Fig 7 |
| JW7298 | *trs120-M1-his5-kanMX6 GFP-bgs1-leu1^+^ bgs1Δ::ura4^+^ rlc1-tdTomato-*  *natMX6 ade6-M210 leu1-32 ura4* | Fig 7 |
| JW7175 | *kanMX6-Pypt3-mEGFP-ypt3 Trs120-tdTomato-natMX6 ade6-210 leu1-32*  *ura4-D18* | Fig 8 |
| JW7345 | *h^+^ kanMX6-Pypt2-mEGFP-ypt2 ade6-210 leu1-32 ura4-D18 +* pSM925  [pREP41-tdTomato-ypt3] | Fig 8; S5 Fig |
| JW7166 | *kanMX6-Pypt3-mEGFP-ypt3 rlc1-tdTomato-natMX6 ade6-M210 leu1-32 ura4-D18* | Fig 8; S5 Fig |
| JW7237 | *kanMX6-Pypt3-mEGFP-ypt3 bgs4∆::ura4^+^ Pbgs4^+^::RFP-bgs4^+^-leu1^+^*  *ade6-M210 leu1-32 ura4-D18* | S5 Fig |
| JW7284 | *kanMX6-Pypt2-mEGFP-ypt2 trs120-tdTomato-natMX6 ade6-210 leu1-32*  *ura4-D18* | S5 Fig |
| JW6836 | *h^-^ rlc1-mEGFP-kanMX6 ade6-M210 leu1-32 ura4-D18 +* pSM925  [pREP41-tdTomato-ypt3] | S5 Fig |
| JW7130 | *kanMX6-Pypt3-mEGFP-ypt3 ade6-210 leu1-32 ura4-D18* | S6 Fig |
| JW7155 | *trs120-ts1-his5-kanMX6 kanMX6-Pypt3-mEGFP-ypt3 ade6 leu1-32 ura4* | S6 Fig |
|  |  |  |
| JW6704 | *h^-^ trs120-tdTomato-natMX6 ade6-210 leu1-32 ura4-D18* | S6 Fig |
| JW6915 | *ypt3-i5 trs120-tdTomato-natMX6 ade6 leu1-32 ura4-D18* | S6 Fig |
| JW6548 | *h^+^ GFP-syb1-kanMX6 rlc1-tdTomato-natMX6 ade6 leu1-32 ura4-D18* | S6 Fig |
| JW6935 | *ypt3-i5 GFP-syb1-kanMX6 rlc1-tdTomato-natMX6 ade6 leu1-32 ura4-D18* | S6 Fig |
| JW6916 | *ypt3-i5 ade6 leu1-32 ura4-D18* | S6 Fig |
| JW7035 | *h^-^ trs120-M1-his5-kanMX6 his5Δ ade6-M210 leu1-32 ura4* | S6 Fig |
| JW81 | *h^-^ ade6-210 ura4-D18 leu1-32* | S6 Fig; [2] |
| JW7036 | *h^-^ trs120-ts1-his5-kanMX6 his5Δ ade6-M210 leu1-32 ura4* | S6 Fig |
| JW7125 | *ypt3-i5 trs120-ts1-his5-kanMX6 ade6-M210 leu1-32 ura4* | S6 Fig |
| JW6968 | *tom20-GBP-hphMX6 trs120-3GFP-kanMX6 bgs4∆::ura4^+^*  *Pbgs4^+^::RFP-bgs4^+^-leu1^+^ ade6-M210 leu1-32 ura4-D18* | Fig 9 |
| JW7147 | *tom20-GBP-hphMX6 trs120-3GFP-kanMX6 ade6-M210 leu1-32 ura4-D18*  *+* pSM925 [pREP41-tdTomato-ypt3] | Fig 9 |
| JW7235 | *tom20-GBP-hphMX6 kanMX6-Pypt3-mEGFP-ypt3 bgs4∆::ura4^+^*  *Pbgs4^+^::RFP-bgs4^+^-leu1^+^ ade6-M210 leu1-32 ura4-D18* | Fig 9 |
| JW6918 | *tom20-GBP-hphMX6 trs120-3GFP-kanMX6 sec3-tdTomato-hphMX6*  *ade6-210 leu1-32* | Fig 9 |
| JW7149 | *tom20-GBP-hphMX6 trs120-3GFP-kanMX6 trs130-tdTomato-natMX6 ade6-M210 leu1-32 ura4-D18* | S7 Fig |
| JW6924 | *tom20-GBP-hphMX6 trs120-3GFP-kanMX6 ade6-M210 leu1-32 ura4-D18* | S7 Fig |
| JW7151 | *tom20-GBP-hphMX6 trs130-tdTomato-natMX6 ade6-M210 leu1-32 ura4-D18* | S7 Fig |
| JW6969 | *tom20-GBP-hphMX6 bgs4∆::ura4^+^ Pbgs4^+^::RFP-bgs4^+^-leu1^+^ ade6-M210*  *leu1-32 ura4-D18* | S7 Fig |
| JW7148 | *tom20-GBP-hphMX6 trs8502-3GFP-kanMX6 ade6-M210 leu1-32 ura4-D18*  *+* pSM925 [pREP41-tdTomato-ypt3] | S7 Fig |
| JW6901 | *h^-^ tom20-GBP-hphMX6 ade6-M210 leu1-32 ura4-D18* | Fig 10 |
| JW6924 | *tom20-GBP-hphMX6 trs120-3GFP-kanMX6 ade6-M210 leu1-32 ura4-D18* | Fig 10 |
| JW6976 | *tom20-GBP-hphMX6 sec3-GFP-kanMX6 ade6-M210 leu1-32 ura4-D18* | Fig 10 |
| JW6548 | *h^+^ GFP-syb1-kanMX6 rlc1-tdTomato-natMX6 ade6 leu1-32 ura4-D18* | S1 and S2 Video |
| JW2402 | *psy1Δ::kanMX6 leu1^+^::GFP-psy1 rlc1-tdTomato-natMX6 ade6-M210 ura4* | S3 Video; [1] |
| JW6982 | *myo52-GFP-kanMX6 rlc1-tdTomato-natMX6 ade6 leu1-32 ura4-D18* | S4 Video |
| JW6771 | *fim1-mEGFP-kanMX6 rlc1-tdTomato-natMX6 ade6 leu1-32 ura4-D18* | S5 Video |
| JW6731 | *trs120-3GFP-kanMX6 rlc1-tdTomato-natMX6 ade6-M210 leu1-32 ura4-D18* | S6 and S7 Video |
| JW7166 | *kanMX6-Pypt3-mEGFP-ypt3 rlc1-tdTomato-natMX6 ade6-M210 leu1-32 ura4-D18* | S8 and S9 Video |

^a^Because diploid cells are not stable for long-term storage and we only used freshly made diploid strains for tetrad fluorescence microscopy in Fig 6A and 6B, so only the parental strains are listed.

**References**

1. Ye Y, Lee I-J, Runge KW, Wu J-Q. Roles of putative Rho-GEF Gef2 in division-site positioning and contractile-ring function in fission yeast cytokinesis. Mol Biol Cell. 2012; 23(7):1181-95. Epub 2012/02/03. doi: mbc.E11-09-0800 [pii] 10.1091/mbc.E11-09-0800. PubMed PMID: 22298427; PubMed Central PMCID: PMC3315812.

2. Wu J-Q, Kuhn JR, Kovar DR, Pollard TD. Spatial and temporal pathway for assembly and constriction of the contractile ring in fission yeast cytokinesis. Dev Cell. 2003; 5(5):723-34. Epub 2003/11/07. doi: S1534580703003241 [pii]. PubMed PMID: 14602073.

3. Wang H, Tang X, Liu J, Trautmann S, Balasundaram D, McCollum D, et al. The multiprotein exocyst complex is essential for cell separation in *Schizosaccharomyces pombe*. Mol Biol Cell. 2002; 13(2):515-29. Epub 2002/02/21. doi: 10.1091/mbc.01-11-0542. PubMed PMID: 11854409; PubMed Central PMCID: PMC65646.

4. Coffman VC, Sees JA, Kovar DR, Wu J-Q. The formins Cdc12 and For3 cooperate during contractile ring assembly in cytokinesis. J Cell Biol. 2013; 203(1):101-14. doi: 10.1083/jcb.201305022. PubMed PMID: 24127216; PubMed Central PMCID: PMC3798249.

5. Wu P, Zhao R, Ye Y, Wu J-Q. Roles of the DYRK kinase Pom2 in cytokinesis, mitochondrial morphology, and sporulation in fission yeast. PLoS One. 2011; 6(12):e28000. Epub 2011/12/17. doi: 10.1371/journal.pone.0028000 PONE-D-11-14680 [pii]. PubMed PMID: 22174761; PubMed Central PMCID: PMC3236194.

6. Wang N, Wang M, Zhu YH, Grosel TW, Sun D, Kudryashov DS, Wu J-Q. The Rho-GEF Gef3 interacts with the septin complex and activates the GTPase Rho4 during fission yeast cytokinesis. Mol Biol Cell. 2015; 26(2):238-55. doi: 10.1091/mbc.E14-07-1196. PubMed PMID: 25411334; PubMed Central PMCID: PMC4294672.

7. Jourdain I, Dooley HC, Toda T. Fission yeast Sec3 bridges the exocyst complex to the actin cytoskeleton. Traffic. 2012; 13(11):1481-95. doi: 10.1111/j.1600-0854.2012.01408.x. PubMed PMID: 22891673; PubMed Central PMCID: PMC3531892.

8. Santos B, Martin-Cuadrado AB, Vazquez de Aldana CR, del Rey F, Perez P. Rho4 GTPase is involved in secretion of glucanases during fission yeast cytokinesis. Eukaryot Cell. 2005; 4(10):1639-45. Epub 2005/10/11. doi: 4/10/1639 [pii] 10.1128/EC.4.10.1639-1645.2005. PubMed PMID: 16215171; PubMed Central PMCID: PMC1265894.
